# Polycyclic Aromatic Hydrocarbon-Induced Changes in Bacterial Community Structure under Anoxic Nitrate Reducing Conditions

**DOI:** 10.3389/fmicb.2016.01775

**Published:** 2016-11-08

**Authors:** Sophie-Marie Martirani-Von Abercron, Daniel Pacheco, Patricia Benito-Santano, Patricia Marín, Silvia Marqués

**Affiliations:** Estación Experimental del Zaidín, Department of Environmental Protection, Consejo Superior de Investigaciones CientíficasGranada, Spain

**Keywords:** anaerobic naphthalene biodegradation, nitrate reducing bacteria, 2-methylnaphthalene, *Acidobacteria*, iii1-8, PAHs, compost pile

## Abstract

Although bacterial anaerobic degradation of mono-aromatic compounds has been characterized in depth, the degradation of polycyclic aromatic hydrocarbons (PAHs) such as naphthalene has only started to be understood in sulfate reducing bacteria, and little is known about the anaerobic degradation of PAHs in nitrate reducing bacteria. Starting from a series of environments which had suffered different degrees of hydrocarbon pollution, we used most probable number (MPN) enumeration to detect and quantify the presence of bacterial communities able to degrade several PAHs using nitrate as electron acceptor. We detected the presence of a substantial nitrate reducing community able to degrade naphthalene, 2-methylnaphthalene (2MN), and anthracene in some of the sites. With the aim of isolating strains able to degrade PAHs under denitrifying conditions, we set up a series of enrichment cultures with nitrate as terminal electron acceptor and PAHs as the only carbon source and followed the changes in the bacterial communities throughout the process. Results evidenced changes attributable to the imposed nitrate respiration regime, which in several samples were exacerbated in the presence of the PAHs. The presence of naphthalene or 2MN enriched the community in groups of uncultured and poorly characterized organisms, and notably in the *Acidobacteria* uncultured group iii1-8, which in some cases was only a minor component of the initial samples. Other phylotypes selected by PAHs in these conditions included *Bacilli*, which were enriched in naphthalene enrichments. Several nitrate reducing strains showing the capacity to grow on PAHs could be isolated on solid media, although the phenotype could not be reproduced in liquid cultures. Analysis of known PAH anaerobic degradation genes in the original samples and enrichment cultures did not reveal the presence of PAH-related *nmsA*-like sequences but confirmed the presence of *bssA*-like genes related to anaerobic toluene degradation. Altogether, our results suggest that PAH degradation by nitrate reducing bacteria may require the contribution of different strains, under culture conditions that still need to be defined.

## Introduction

Polycyclic aromatic hydrocarbons (PAHs) are chemicals of particular environmental concern because of their stability, persistence in the environment, and resistance to degradation. Many of them are known to be toxic to various organisms and dangerous for health. They are frequently released into the environment either from natural sources (e.g., hydrocarbon seeps) or as consequence of industrial activities such as the massive transport or synthesis of added value chemicals. For most low molecular weight PAHs, microbial biodegradation is the primary process leading to their complete mineralization. The biodegradation of PAHs by aerobic bacteria has been studied extensively and is well understood (Fuchs et al., 2011). In aerobic environments, oxygen is not only the terminal electron acceptor for bacterial respiration, but is also an essential co-substrate for the activation and cleavage of the aromatic ring. Enzymatic steps invariably involve mono-oxygenases and dioxygenases as key activities for the molecular oxygen-dependent initial attack of the aromatic ring (Doyle et al., 2008). However, the availability of oxygen is often limited in natural environments. In many polluted sites, oxygen can be rapidly consumed in the aerobic biodegradation processes, resulting in a decreasing oxygen concentration gradient whereupon reduction of other electron acceptors becomes energetically favorable. Sediments are a good example of such a situation, where initially microorganisms consume the oxygen that penetrates the first millimeters/centimeters of the sediments. Once the oxygen consumption exceeds its supply, anoxic conditions are established. Below the zone of oxygen-influence, anoxic processes are stimulated: Nitrate, manganese, iron, and sulfate are then sequentially used for anaerobic respiration, if available (Sørensen, 1978; Canfield et al., 1993). A similar pattern is observed in habitats that are under permanent anoxic conditions, such as flooded sediments in marine and fresh-water environments. Bioremediation of PAHs in these conditions becomes challenging. Our current knowledge of the biochemistry of anaerobic degradation of PAHs is restricted to the simplest molecules naphthalene and 2-methylnaphthalene (2MN) in sulfate-reducing bacteria (SRB). To date only two examples of naphthalene degrading SRBs have been described and isolated, one of which is a two-strain consortium (N47) that could not be obtained as a pure isolate (DiDonato et al., 2010; Selesi et al., 2010). In SRB, naphthalene is activated through carboxylation (Zhang and Young, 1997), and the resulting naphtoic acid is further converted to its coenzyme A (CoA) thioester 2-naphtoil-CoA. Further dearomatization of 2-naphtoil-CoA proceeds sequentially for the two aromatic rings: First a 2-naphthoyl CoA reductase, encoded by the *ncr* gene, and then a 5,6-dihydro-2-naphthoyl-CoA reductase, catalyze two sequential 2-electron reduction steps, followed by the final reduction of the resulting 5,6,7,8-tetrahydronaphthoyl-CoA to hexahydro-2-naphthoyl-CoA (DiDonato et al., 2010; Boll et al., 2014; Estelmann et al., 2015). On the other hand, anaerobic degradation of 2MN by SRB requires initial activation through addition of its methyl group to fumarate to render naphthyl-2-methyl-succinic acid, a reaction carried out by a naphthyl-2-methylsuccinate synthase encoded by the *nmsA* gene (Selesi et al., 2010). Naphthyl-2-methyl-succinic acid is further transformed to 2-naphthoil-CoA. To date, these pathways have been biochemically and genetically verified in only one SRB isolate (DiDonato et al., 2010) and in a sulfate-reducing enrichment (Selesi et al., 2010). Only recently SRB microcosms with naphthalene as carbon source could link the enrichment of *Desulfobacterium* strains related to N47 with naphthalene degradation, although the strains were not isolated (Kümmel et al., 2015). Anaerobic degradation of PAHs with nitrate as electron acceptor has been repeatedly observed in environmental samples and microcosm experiments (Al-Bashir et al., 1990; Eriksson et al., 2003; Uribe-Jongbloed and Bishop, 2007; Acosta-González et al., 2013a), but little is known about the degradation mechanism in nitrate reducing bacteria (NRB). Initial reports of naphthalene-degrading isolates closely related to *Pseudomonas stutzeri* and *Vibrio pelagius* (Röckne et al., 2000) could not be reproduced. Despite efforts by different groups to isolate NRB able to degrade PAHs under anoxic conditions, to date no bacterial strain or consortium able to consistently degrade naphthalene using nitrate as terminal electron acceptor has been described.

With the aim of assessing nitrate reduction-dependent PAH degradation in the environment and identifying potential organisms involved in the process, we selected a series of environmental sites that had been exposed to different degrees of hydrocarbon pollution and presented transient or permanent anoxic conditions. Most probable number estimation evidenced the presence of an initial PAH degrading NRB community. Starting from this material, we initiated enrichment cultures under nitrate reducing conditions with naphthalene and its methylated derivative 2MN as the added carbon source. We used culture-dependent and molecular techniques to investigate the effects that incubation with PAHs produced on the natural microbial communities present in the selected environments. We anticipated that exposure to PAHs would result in an altered microbial community structure reflecting both the toxicity of the added aromatic and the opportunity for the biodegradation of a new carbon source under the imposed respiration metabolism. We found that shifting to nitrate as terminal electron acceptor strongly affected the structure of the bacterial community. The presence of naphthalene further disturbed microbial communities and produced a general increase in poorly characterized and uncultured groups.

## Materials and methods

### Sample collection and experimental design

Samples were collected from 5 different sampling sites (Table 1). Two rice-paddy soil samples with different water content were taken in November 2011 from surface soil (0–15 cm depth) in a rice paddy located in Las Cabezas de San Juan (Sevilla, Spain) (37°01′42N 5°58′49W), close to some fuel oil leaks. One was obtained from the more aqueous top layer (upper 5 cm) (RPW, Rice-Paddy Water) and the other one from the bottom layer (~5–10 cm) (RPS, Rice-Paddy Soil). A third rice-paddy sample (RPCal) was collected in December 2011 from the surface (0–15 cm) of a rice-paddy in Calasparra (Murcia, Spain) (38°14′44N 1°41′32W). The activated sludge (AS) and composting pile (CP) samples were collected in July 2010 from the CEPSA oil refinery in La Rábida, Huelva (37°34′15N 0°55′30W). The marine sediment sample (MS) was collected by scuba divers at 5–6 m below the water surface from Figueiras beach (Atlantic Islands, Spain) in June 2005 (42°13′31N 8°53′59W) using 5 cm diameter cores inserted in the sediment. The sediment column between 2 and 35 cm was mixed and used in this study. Anoxic black sediments (0–10 cm in depth) from the athalassohaline lagoon of Fuente de Piedra (FdP) (Málaga, Spain) were collected close to the shore (50 cm below water surface) in March 2012 (37°05′07N 4°47′05W).

**Table 1:** **Characterization of the samples used in this study**.

**Sample Name**	**Sample type**	**Description**	**Location**	**Coordinates**	**Water^a^ content (%)**	**Organic matter (%)**	**Nitrate (μM)**	**Sulfate (mM)**	**Total hydrocarbons^c^ (ug/kg)**	
									**Aliphatic**	**Aromatics**
RPCal	Rice pad	Soil	Calasparra (Murcia)	38°14′44N 1°41′32W	18.43 ± 0.23	2.50 ± 0.13	416.65 ± 5.2	0.04 ± 0.004	2990	857.0
RPW	Rice pad	Overlaying muddy water	Sevilla	37°01′42N 5°58′49W	74.85^b^	3.37^b^	14.96 ± 5.1	0.32 ± 0.004	3990	521.0
RPS	Rice pad	Soil	Sevilla	37°01′42N 5°58′49W	5.6 ± 0.0039	4.14 ± 0.08	3.00 ± 0.7	0.09 ± 0.009	3890	460.0
AS	Activated sludge	Mud	Huelva	37°34′15N 0°55′30W	1975.04 ± 390	63.59 ± 0.14	12.65 ± 1.4	0.64 ± 0.02	45150	888.5
CP	Compost pile	Soil	Huelva	37°34′15N 0°55′30W	0.68 ± 0.06	0.94 ± 0.03	501.72 ± 8.9	2.37 ± 0.20	1760	745.1
FdP	Athalassohaline lagoon	Sediment	Fuente de Piedra (Málaga)	37°05′07N 4°47′05W	107.58 ± 6.39	5.87 ± 0.59	0	26.48 ± 3.15	26400	117.0
MS	Marine sediments	*Prestige* oil spill-affected sandy sediments	(Pontevedra)	42°13′31N 8°53′59W	24.74 ± 0.22	0.58 ± 0.02	117.79 ± 4.7	3.15 ± 0.14	1740	95.0

a *Gravimetric, % of dry weight*.

b *The sample duplicate was lost*.

c *The detailed hydrocarbon composition of the samples can be found in Tables S3A,B*.

### Culture conditions, enrichment, and isolation procedures

For the cultivation of nitrate-reducing bacteria (NRB) 25 g of samples were transferred to 120 ml serum bottles containing 75 ml of non-reduced Widdel mineral medium, modified according to Darley et al. (2007): The medium was buffered with 30 mM 3-(N-morpholino)propanesulfonic acid (MOPS) instead of bicarbonate, prepared in a nitrogen atmosphere and sealed with Teflon-lined 1 cm thick stoppers. The basal freshwater medium used for the soil and sludge samples contained 1.0 g NaC1 and 0.4 g MgCl·6H_2_O per liter, whilst the marine medium used for the marine and lagoon sediments contained 20.0 g NaC1 and 3.0 g MgCl_2_ per liter. The cultures were set up as follows: Briefly, after autoclaving, the medium was cooled in a sterile nitrogen atmosphere by flowing pure nitrogen gas through it for at least 20 min, and supplemented with 5 mM NaNO_3_ as electron acceptor, 1 mM Na_2_SO_4_ as the sulfur source, 1 ml of a vitamin solution, 1 ml of the trace element solution SL10, 1 ml selenite-tungstate solution, and 30 mM MOPS buffer, pH 7.2 (Darley et al., 2007), and dispensed in 120 ml serum bottles flushed with sterile nitrogen gas. Naphthalene and 2-methylnaphthalene (2MN) were dissolved at 20 g l^−1^ in sterile anoxic 2,2,4,4,6,8,8-heptamethylnonane (HMN) that served as an inert carrier phase to reduce the PAH concentration in the water phase. The resulting suspension (2 ml) was then added to the serum bottles as an overlay using a nitrogen-flushed syringe. A control bottle without added aromatic compounds in the HMN phase was prepared for each sample. Then the appropriate amount of inoculum was added and the cultures were sealed with Teflon-lined 1 cm thick stoppers fixed with aluminum crimp seals. The cultures were incubated in the dark at 28°C without shaking. To determine growth, the cultures were monitored periodically for nitrite produced from nitrate respiration (Snell and Snell, 1949), or for nitrate consumption by ion chromatography. After 3 months' of incubation, the medium above soil/sediment particles and the organic layer were removed and replaced by fresh mineral medium and the corresponding carbon source in HMN. This operation was repeated twice every 3 months, and after an additional 2-month incubation samples were taken for pyrosequencing. Further transfers (every 6 months) were made by inoculating 10% (v/v) of the cultures into fresh medium. At several stages bacterial strains were isolated under anoxic conditions, either using serial agar dilutions (agar shakes) prepared in a nitrogen atmosphere (Widdel and Bak, 1992) and overlaid with aromatic hydrocarbons in HMN, or in standard agar Petri dishes prepared and incubated in a nitrogen atmosphere with naphthalene provided in the vapor phase as crystals on the Petri dish cover placed up-side-down.

### Most probable number enumeration of denitrifying bacteria

Nitrate reducing PAH-degrading bacterial populations were enumerated using the most-probable number (MPN) assay in 10-fold serial dilutions. The medium was prepared and dispensed as described above. Naphthalene, 2-methylnaphthalene, naphtoic acid and anthracene were added to the tubes from stock diethyl ether solutions to reach a final concentration of 0.025, 0.028, 0.035, and 0.035‰, respectively, and the solvent was evaporated before dispensing the medium. Acetate was used as a positive control to a final concentration of 5 mM, and controls with no added carbon source were included. The minimal medium containing 5 mM sodium nitrate as the electron acceptor was purged with sterile nitrogen gas and 9 ml were poured into oxygen-free tubes. Growth of NRB was measured as the nitrite produced from nitrate respiration as described above. For each sample, triplicate tubes were inoculated with 1 ml of the corresponding dilution in 10-fold serial dilutions. The tubes were incubated at 28°C in the dark and growth was checked during 6 months. The data are the result of three replicate per sample within the 95% confidence interval.

### Chemical analysis

All determinations were carried out in duplicate just before starting the enrichments. Soil moisture was determined as follows: Samples (5 g) were dried to constant mass in an oven at 105°C. The difference in mass before and after the drying process was used to determine the dry matter and the water content. The loss-on-ignition method was used to determine the organic matter content (Schumacher, 2002). The concentration of soil-available nitrate and sulfate was measured by ion chromatography (IC) using a Metrohm-761 Compact Ion Chromatograph with a Metrosep A Supp 4–250 column with chemical suppression (50 mM H_2_SO_4_). The eluent was an aqueous sodium carbonate/bicarbonate solution prepared by mixing NaHCO_3_ and NaCO_3_ to a final concentration of 1.7 and 1.8 mM, respectively. Samples (1 g) were extracted with 5 ml of deionized water by mechanical agitation for 16 h and then analyzed by IC. The detection limit for ions was 0.5 ppm. The detection limit for nitrate determination with this method was 0.5 ppm, and the associated error was 5%. For aliphatic and aromatic hydrocarbon compounds determination we used duplicate 5 g aliquots of soil/sediment samples. Prior to the extraction 100 ppb of the Mix 37 (manufactured by Dr. Ehrenstorfer) and 15 ppm of the 5-alpha-cholestane (Aldrich) were added to each sample as internal standards. A volume of 10 ml of a hexane/acetone mixture (2:1) was added to the samples, and the mixture was shacked and sonicated twice during 5 min. Next 20 ml of saturated NaCl were added to the suspensions. The supernatants were collected and passed through a BOND ELUT TPH 500 mg Na_2_SO_4_ column (Varian) previously conditioned with 3 ml of hexane to separate the aliphatic and the aromatic fractions. The aliphatic fraction was first collected and the BOND ELUTE TPH columns were dried completely. The aromatic fraction was next eluted with 6.5 ml of dichloromethane. The extracts were concentrated by evaporation under a gentle nitrogen flow and the hydrocarbons were determined at the Scientific Instrumentation Service of the EEZ (CSIC), Granada, Spain, in a Varian 450-GC GC gas chromatographer system coupled to a Varian 240-MS ion-trap mass spectrometer with MS Workstation software, equipped with the TG-5SilMS column (30 m × 0.25 mm × 0.25 μm) and a CTCGCpal autosampler. Data were acquired and processed using Varian MS Workstation 6.9.1. Helium was used as carrier gas at a flow rate of 1 ml/min. The injected volume was 1 μl (splitless mode). The oven temperature started at 50°C and was raised at 15°C/min up to 150°C. A new ramp of 6°C/min was started up to 300°C, when the temperature was held for 10 min. Injector and detector temperatures were set at 300 and 290°C respectively. Compound identification was performed using the NIST08 library included in the MS Workstation software 6.9.1 and the information provided by the standards injected under the same conditions. The standards used in the chromatography were the alkane mixture DRH-008S-R2 y DRH-001S, the PAH mixture Mix 37 y Mix 9 (manufactured by Dr. Ehrenstorfer), 1-Chlorooctadecane and 5-alpha-cholestane, and the pure compounds naphthalene, 1-methylnaphthalene, 2MN, 1-4-5-trimethylnaphthalene, 1-4-6-7-tetramethylnaphthalene, dibenzothiophene, phenanthrene, and anthracene. The average PAH extraction efficiency obtained with our extraction method was 64(± 18)%.

### Total DNA extraction, PCR amplification, and pyrosequencing library construction

DNA extraction was done following a modified version of the SDS-based method developed by Zhou et al. (1996) except that in the third extraction three freeze-thaw cycles were included before the incubation at 65°C. Supernatants from the three extractions were combined and extracted first with an equal volume of phenol-chloroform-isoamylalcohol (25:24:1, v/v) and then with chloroform-isoamylalcohol (24:1, v/v), and precipitated overnight at room temperature with 0.6 volume of isopropanol. Nucleic acids were recovered by centrifugation (7000 rcf/45 min), washed with cold 70% ethanol, resuspended in 100 μl of sterile deionized water and stored at −20°C. Nucleic acid quantity and purity were determined with a NanoDrop ND-1000 Spectrophotometer (NanoDrop Technologies). A multiplex pyrosequencing amplicon approach was used for the characterization of the bacterial communities. The PCR amplifications of the hypervariable V1–V3 region of the 16S rRNA gene were carried out using the bacterial universal primers 6F and 532R containing 5′ tags with multiplex identifier and sequencing adapters (Tables S1A,B). PCR amplifications were performed in 50 μl reactions containing 1x PCR Buffer (Biorad), 200 μM dNTPs (Roche), 0.5 μM of each primer (Roche), 1 U of iProof™ High-Fidelity DNA Polymerase (Biorad) and 20 ng of target DNA. The PCR program consisted of an initial denaturation step at 98°C for 30 s, followed by 25 cycles at 98°C for 10 s, 50°C for 20 s, and 72°C for 30 s, with a final extension at 72°C for 5 min. The PCR fragments were purified using the PCR Purification Kit (Qiagen) and checked on 1.5% agarose gel. Amplicon products with adapters and barcodes were quantified using Qubit™ fluorometer (Invitrogen), pooled at an equimolar ratio and sequenced using a 454 Titanium amplicon sequencing kit and a Genome Sequencer FLX 454 at either Citius (University of Seville) or Macrogen (Korea).

### Functional gene amplification

Gene fragments of *bssA, ncr*, and *nmsA* were amplified by PCR using available primer sets (Table S2). For *bssA* gene amplification we used 7772f and 8546r primers (Winderl et al., 2007) and cycling conditions as described by Acosta-González et al. (2013b). *Thauera aromatica* K172 DNA was used as positive control. The *nmsA* gene was amplified using different primer sets (7768f, 7363f, 7374f, and 8543r; (von Netzer et al., 2013) and the following cycling conditions: 1 min of initial denaturation (98°C), 30–35 amplification cycles of (10 s at 98°C, 20 s at 58°C, 30 s at 72°C) and 5 min of final extension (72°C). PCR conditions for the *ncr* gene amplification comprised initial denaturation at 98°C for 1 min, followed by 30 amplification cycles (20 s at 98°C, 20 s at 55°C, 15 s at 72°C), and a final extension step at 72°C for 5 min. Genomic DNA from NaphS2 strain was used as a positive control. All PCR reactions were performed in 50 μl containing 1x PCR Buffer (Biorad), 200 μM dNTPs (Roche), 0.5 μM of each primer (Sigma), 1 U of iProof™ High-Fidelity DNA Polymerase (Biorad) and 10 to 20 ng of target DNA. The appropriately sized amplicons were purified using the Gel Extraction Kit (Qiagen) and cloned in pCR2.1 (TA Cloning Kit, Invitrogen) according to the manufacturer's instructions, and 16 positive clones from each sample were selected for Sanger sequencing (IPBLN López Neyra, CSIC, Granada, Spain). Sequence analysis was performed as described previously (Acosta-González et al., 2013b).

### 16S rRNA gene amplification

PCR amplification of the 16S rRNA gene to identify the isolated strains was performed with the bacterial universal primers GM3F and GM4R (Muyzer et al., 1995). PCR reactions were carried out in 50 μl reactions as previously described (Acosta-González et al., 2013b). The amplified fragments were cloned in pGEM-T (Promega) according to the manufacturer instructions. Positive clones were checked by PCR with pUC/M13F and pUC/M13R primers and were Sanger-sequenced as above. The generated chromatograms were analyzed and edited with the Chromas (Technelysium) and DNA Baser (Heracle Biosoft) softwares for quality checking, vector trimming and sequence assembly. Phylogenetic analysis were done with the ARB package (Ludwig et al., 2004) using the online SINA alignment service and Silva database version SSU Ref 119.

### Data analysis

The 454 bacterial 16S sequences were analyzed using the Quantitative Insights Into Microbial Ecology (QIIME) v. 1.7.0 pipeline (Caporaso et al., 2010). A total of 32 samples were analyzed. We first performed the sample demultiplexing, primer removal and quality-filtering. Briefly, sequences with lengths <150 bp, ambiguous bases >0, homopolymers >6, primer mismatches, and average quality scores <50 were removed. Reads were checked for chimeras using the ChimeraSlayer algorithm (Haas et al., 2011). All chimeras and singletons were removed before further analysis. The clustering method was used to assign similar sequences to operational taxonomic units (OTUs) at a 97% similarity threshold. A representative sequence from each OTU was annotated with PyNAST (Caporaso et al., 2010). The taxonomic assignment of OTUs was performed using the RDP Classifier. A variable number of sequences were obtained per sample; to avoid sampling size effects, the number of reads was normalized to 1500 for each sample. Otherwise the non-rarefied OTU table was used. Alpha and Beta diversity analyses, rarefaction curves, Chao1 richness estimator, Shannon diversity index and Good's sample coverage were calculated using QIIME v. 1.7.0 pipeline. Principal coordinate analysis (PCoA) was performed using the subsampled data to detect microbial community differences on the basis of weighted and unweighted UniFrac distance metrics (Lozupone and Knight, 2005). Jackknife resampling was used to assess the stability of the PCoA analysis. Hierarchical clustering was conducted to group the communities of different samples using the unweighted pair group method with arithmetic mean (UPGMA). Differences in the relative abundance among treatments were calculated by two-sided *t*-tests. To construct the phylogenetic trees of iii1-8 sequences we used the Neighbour-Joining method. Bootstrap test (1000 replicates) was used to calculate the percentage of replicate trees in which the associated taxa clustered together. The evolutionary analyses were conducted in MEGA5 (Tamura et al., 2011).

### Nucleotide sequence accession numbers

The 454 pyrosequencing raw reads have been deposited in the NCBI short-reads archive database (accession number SRP077784). The partial *bssA* gene sequences obtained from the clone libraries have been deposited in GenBank under accession numbers KX455933 to KX456036). The 16S rRNA gene clone library sequences have been deposited in GenBank under accession numbers KX417378 to KX KX417406.

## Results and discussion

### Presence of a nitrate-reducing, PAH-degrading bacterial community in environmental samples

**Figure 1:**
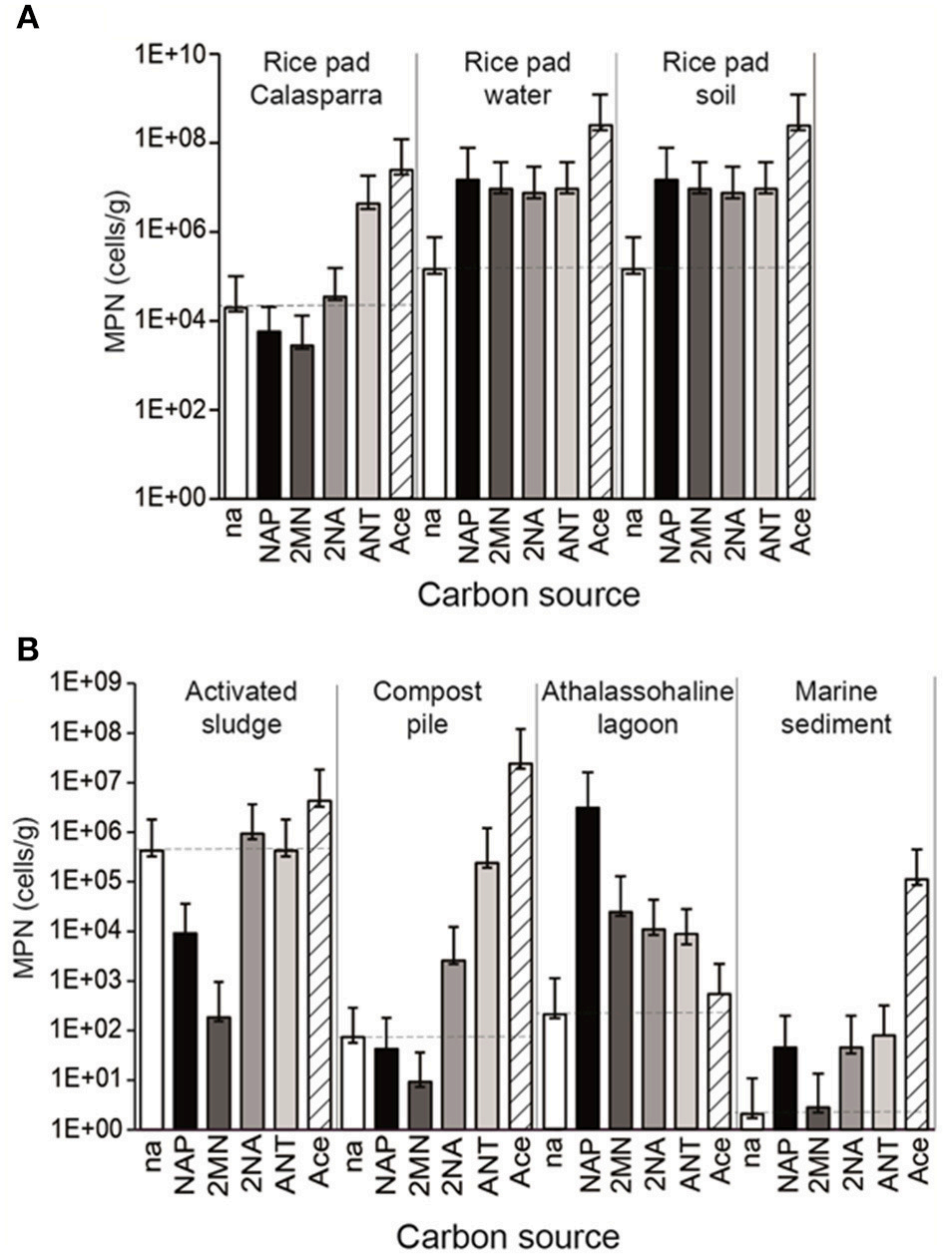
**MPN enumeration in the environmental samples of nitrate reducing bacteria able to grow on naphthalene (NAP), 2-methyl-naphthalene (2MN), 2-naphtoic acid (2NA), anthracene (ANT), acetate (Ace), and with no added carbon source (na)**. Counts were made in triplicate. Numerical values (95% C.I.) can be found in supplementary Table S4. Dotted lines indicate the basal growth with no added carbon source. **(A)** Rice paddy samples **(B)** Oil refinery and sediment samples.

To assess the presence of PAH-degrading bacterial communities, we selected a range of environments that had been exposed to different degrees of hydrocarbon pollution (Table 1). Water and soil from two different rice-paddy fields (RPCal, RPS, RPW), sediments from the Fuente de Piedra athalassohaline lagoon in southern Spain (FdP), marine sediments affected by an oil spill (MS), activated sludge from an oil refinery (AS), and its resulting composting pile (CP) were sampled and analyzed for total organic matter, electron acceptors and hydrocarbon content, (Table 1; Tables S3A,B). The concentration of PAHs in the initial samples ranged from 95 μg/kg in the MS sample to 888 μg/kg in the AS sample, and was also considerable high in the rice-paddy soil from Calasparra (RPCal) (857 μg/Kg) (Table S3B). These values were in the range of those generally found in industrial areas and agricultural soils (Nadal et al., 2004). MPN enumeration of NRB able to grow with different carbon sources showed that acetate, which was our control as a simple carbon source utilizable by most organisms harboring a TCA cycle, gave the highest counts of NRB in all samples except FdP (Figure 1). As expected, values were highest in the soil samples (in the range of 10^8^ cells/g) and lowest in the marine and lagoon sediments (10^3^–10^5^ cells/g). We observed that the intrinsic OM present in the samples allowed the basal growth of a significant bacterial population, as deduced from MPN values of the unamended control cultures (with no added carbon source) included in the analysis. This was especially true in the AS sample (above 10^5^ cells/g), which also showed the highest values of OM content (Table 1), and in the rice-paddy samples (10^4^–10^5^ cells/g), probably because of a better adaptation of the community to nitrate respiration in this habitat. However, above these basal values, we were able to detect the presence of an autochthonous NRB community capable of growing with naphthalene as carbon source in some of the samples, which reached values as high as 10^7^ cells/g in RPS and RPW samples, 2 × 10^6^ cells/g in the FdP sediments, and almost 10^2^ cells/g in the MS sample, as previously observed (Acosta-González et al., 2013a) (Figure 1, Table S4). These samples also included important communities able to grow with 2-methylnaphthalene (2MN) (except for MS), 2-naphtoic acid (2NA), and anthracene (ANT). In addition, the presence of a significant anthracene degrading NRB community was detected in the rice-paddy and compost-pile samples. In this latter sample we were also able to enumerate a 2NA degrading community, although the MPN of naphthalene and 2MN degrading NRB were equal or below the control values. Surprisingly, in the activated sludge the bacterial counts on all the carbons sources gave values similar or below the basal growth on the sample intrinsic OM. Especially naphthalene and 2MN seemed to produce a toxic effect on the bacterial communities present in the sludge, which diminished by 2 and 3 orders of magnitude, respectively, with respect to the absence of any added carbon source. Although the presence in natural samples of PAH-degrading nitrate reducing communities has been described in different environments (Mihelcic and Luthy, 1988; Al-Bashir et al., 1990; Eriksson et al., 2003; Uribe-Jongbloed and Bishop, 2007), the microorganisms involved in the process have not been identified and the reproducibility of the observations has been questioned (Meckenstock et al., 2016). However, our results suggest that a bacterial community able to utilize PAHs as carbon source with nitrate as terminal electron acceptor was present in some of the samples.

### Bacterial community structure in the selected environments

Enrichment cultures were initiated with the above described environmental samples, nitrate as terminal electron acceptor and HMN-dissolved naphthalene or 2MN as carbon sources. Parallel cultures only supplemented with HMN were run as controls. After 6 months enrichment with two culture transfers (See Materials and Methods Section), samples were taken to analyze changes in the bacterial communities using 16S rRNA gene V1–V3 region pyrosequencing. DNA from duplicate samples of the starting material was analyzed in parallel.

**Figure 2:**
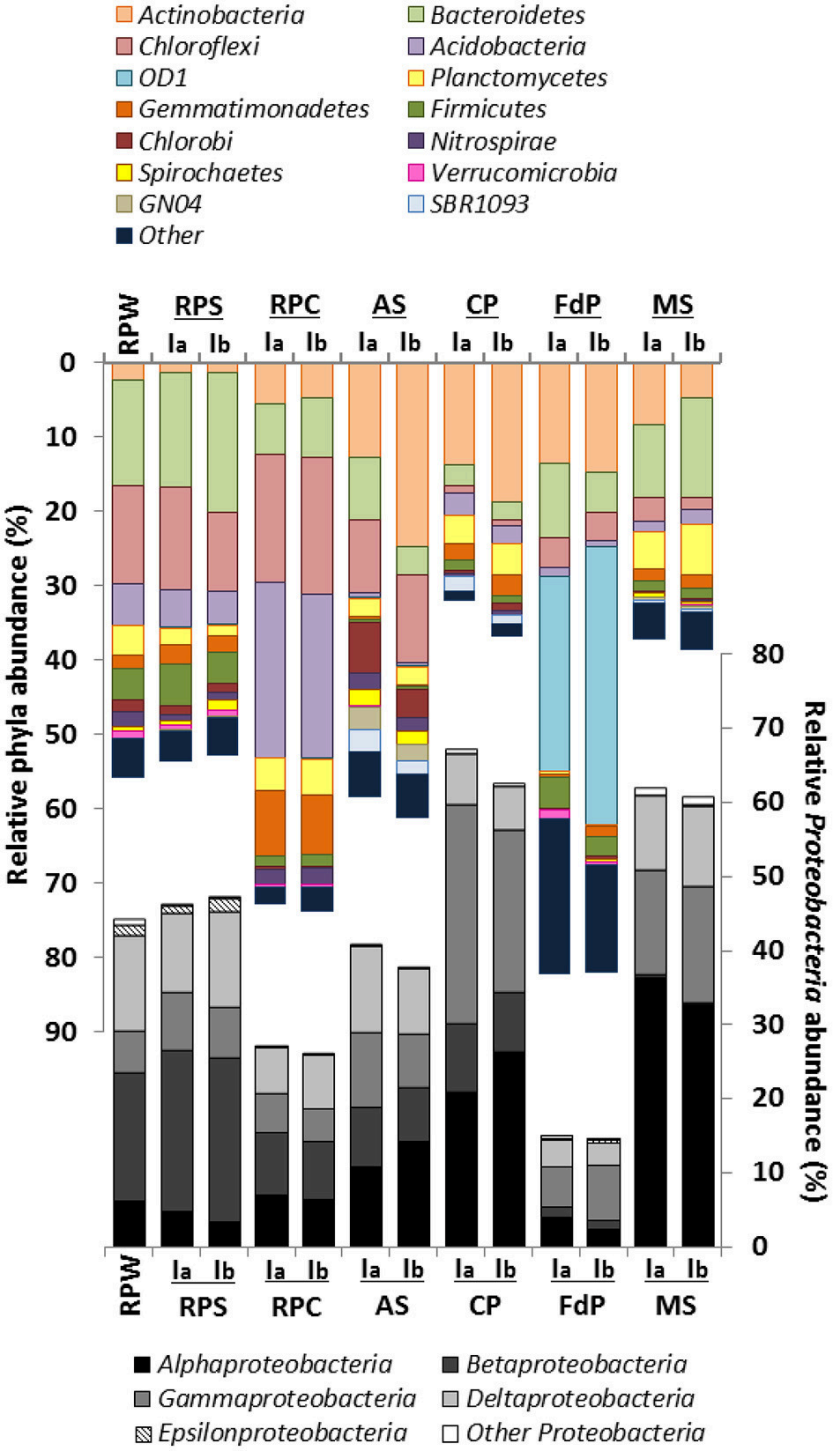
**Cumulative plot of bacterial phyla detected in the initial environmental samples**. Samples were analyzed in duplicate (labeled Ia and Ib) except for RPW, for which one of the samples was lost. RPS, rice-paddy soil; RPW, rice-paddy water, RPCal, rice-paddy Calasparra; AS, activated sludge; CP, compost pile; MS, marine sediment, FdP, Fuente de Piedra athalassohaline lagoon. Numerical values can be found in Table S5.

A total of 262,601 sequences from the 32 amplicon-libraries were obtained after applying all quality filters and deleting dimers and singletons (Table 2). The read numbers ranged between 1907 and 16,246, with an average of 8206 sequences per sample. The rarefaction curves (Figure S1) and the Good's sample coverage estimator, which gave value between 75.54% (RPC-2MN) and 99.19% (CP-2MN), suggested that sampling depth was sufficient to estimate the microbial diversity in all the samples (Table 2). The community structures of the initial samples varied considerably between environment types, as expected, and were in agreement with the bacterial distribution expected for each ecosystem. The observed OTU number and diversity indices were reproducible between replica (Table 2) and indicated that the bacterial richness and diversity were highest in the rice field soils, reflecting the known complexity of soil ecosystems, which are amongst the most diverse environments (Torsvik et al., 2002). The number of OTUs was in the same range in the marine sediment samples, in accordance with global studies showing that sediments are more phylogenetically diverse than any other habitat (Lozupone and Knight, 2007), and reached intermediate values in the remaining samples. Figure 2 shows that the community structure of the initial environments was dominated by the phylum *Proteobacteria* in all cases except FdP, followed, in different proportions, by *Bacteroidetes, Chloroflexi*, and *Actinobacteria*. Interestingly, the FdP samples were dominated by the uncultured candidate phylum *Parcubacteria* (former OD1 candidate division), and the *Proteobacteria* only represented 15% of this community (Table S5).

**Table 2:** **Comparison of OTU number, diversity, evenness indices, and coverage for the different samples**.

**Sample^a^**	**NS^b^**	**OTUs^c^**	**OTUs (1500)^d^**	**Chao1 (1500)^e^**	**Shannon**	**Coverage^f^(%)**
RPW-I_a_	3026	1210	791.6	1748.4	9.5	80.32
RPW-N	5920	1072	530.5	1065.4	8.4	92.79
RPW-2MN	7584	1148	490.2	1064.8	8.1	94.04
RPS-I_a_	6425	1706	745.1	1818.2	9.5	89.31
RPS-I_b_	5962	1631	744.7	1763.2	9.5	89.38
RPS-N	15953	1710	492.2	1146.6	8.0	96.06
RPS-2MN	8753	1412	550.6	1234.9	8.4	93.51
RPCal-I_a_	4276	1388	786.9	1611.0	9.6	87.11
RPCal-I_b_	4115	1443	809.5	1690.1	9.7	85.95
RPCal-N	6662	1620	716.4	1569.4	9.4	90.54
RPCal-2MN	1907	853	738.6	1649.4	9.0	75.54
RPCal-HMN	3164	1104	712.6	1448.8	9.2	83.67
AS-I_a_	7821	686	317.1	621.0	6.8	96.82
AS-I_b_	6787	635	297.5	570.4	6.4	96.12
AS-N	16246	966	345.0	609.8	7.5	98.29
AS-2MN	5781	674	354.7	665.6	7.3	95.37
AS-HMN	13257	923	358.5	658.5	7.5	97.69
CP-I_a_	9195	694	311.9	584.8	7.0	97.52
CP-I_b_	5029	531	313.1	528.8	7.1	96.11
CP-N	8278	206	94.3	166.7	3.2	98.99
CP-2MN	8772	214	103.7	187.6	3.4	99.19
CP-HMN	8608	302	163.1	237.5	5.2	98.98
FdP-I_a_	6202	591	304.8	592.8	6.3	96.78
FdP-I_b_	3542	468	319.4	537.8	6.7	95.14
FdP-N	5252	743	386.2	841.2	6.6	94.11
FdP2-2MN	14660	1097	338.1	798.0	5.7	97.53
FdP-HMN	13789	1179	381.7	909.5	6.4	97.11
MS-I_a_	7711	1219	500.2	1082.7	8.2	94.18
MS-I_b_	8159	1179	487.9	1140.2	7.8	93.96
MS-N	10818	414	178.5	299.3	5.9	98.53
MS-2MN	16260	484	180.7	306.6	5.6	98.92
MS-HMN	12687	446	176.0	318.4	5.6	98.56

a *I_a_ and I_b_ refer to the two replica of the initial samples of each environment, except for RPW-I, where one of the replica was lost*.

b *Number of sequences for each library filtered for chimera and singletons*.

c *OTU numbers calculated with all sequences at the 3% distance level*.

d *OTU numbers calculated for a randomized subset of 1500 reads per sample at the 3% distance level*.

e *Chao index calculated with 1500 subsampled sequences*.

f *Good's sample coverage estimator*.

The community structure in the three rice-paddy samples was similar: The sequences classified as *Proteobacteria* (26.5–46.6%), *Chloroflexi* (10–17.8%), *Acidobacteria* (4.7–22.8%), *Bacteroidetes* (7.4–17.1%), *Gemmatimonadetes* (2–8.4%), *Planctomycetes* (1.9–4.6%), *Actinobacteria* (1.3–5.2%), *Firmicutes* (1.5–4.9%), and *Nitrospirae* (2–2.1%) accounted for 84–96% of all sequences. Although the abundance of dominant phyla in soil environments has been shown to be highly variable, and many factors can affect phyla distribution, the predominance of these groups in the paddy soils was expected, as these phylotypes have been described as common inhabitants of agricultural soils (Lopes et al., 2014). Members of these groups have been shown to account for 90% of the microbial population in these habitats (Janssen, 2006).

The AS and CP samples originated from early and final steps, respectively, in the wastewater treatment of an oil refinery. In oil refineries, the wastewater influent can differ daily in terms of concentrations and composition of pollutants, which results in a high variability of the community distribution in these habitats (Hu et al., 2013). In the AS sample the community was dominated by *Proteobacteria* (39.1%), followed by *Actinobacteria* (18.7%), *Chloroflexi* (10.7%), *Bacteroidetes* (6.2%), *Chlorobi* (5.3%), *Spirochaetes* (2%), *Planctomycetes* (2.3%), *SBR1093* (2.4%), and *GN04* (2.6%), which made up about 90% of the libraries. *Proteobacteria* also represented the predominant phylum in different activated sludge samples, suggesting that these organisms are involved in biodegradation processes and in the removal of organic pollutants such as aromatic compounds (Wagner and Loy, 2002; Yang et al., 2011; Silva et al., 2012). In contrast, in the CP sample the *Proteobacteria* were twice as abundant, especially the *Gammaproteobacteria* class. Furthermore, members of the *Planctomycetes* (3.9%) and especially *Acidobacteria* (2.9%), which were below 1% in the AS sample, were more represented in this sample. On the other hand, some groups were less abundant, such as *Bacteroidetes* (2.6%), and especially *Chlorobi* (0.7%), and *Chloroflexi* (0.8%), which were almost absent.

In the FdP sediment samples the most abundant phyla was the candidate division *OD1* (*Parcubacteria*) (31.8%), followed by the *Proteobacteria* (14.9%), *Actinobacteria* (14.1%), *Bacteroidetes* (7.7%), and *Firmicutes* (3.5%). Although this type of environment is of great interest, its prokaryotic diversity and especially that of the anoxic sediments, has been poorly studied. In a number of sites with similar characteristics, the most prominent groups were *Proteobacteria, Bacteroidetes*, and *Firmicutes* (Demergasso et al., 2004; Jiang et al., 2006; Mesbah et al., 2007). The phylum *Parcubacteria*, with no cultivated member to date, has been detected globally in both aquatic and terrestrial habitats and mostly in anoxic environments (Elshahed et al., 2005; Peura et al., 2012; Rinke et al., 2013). The dominance of *Parcubacteria* in this type of lagoon has not yet been reported and its ecological role is unclear, although members of this phylum are commonly identified in anoxic environments and sediments. The *Parcubacteria* are characterized by the small size of their genome, with a large number of unique genes (Nelson and Stegen, 2015). Analysis of the genomes sequenced so far after single genome amplification suggests poor mechanisms for energy and nutrient conservation (hence the name *Parcubacteria*), the absence of genes for the tricaboxylic acid cycle, electron transport and amino acids, nucleotides, vitamins, and lipids biosynthesis (Wrighton et al., 2012). This paucity of metabolic functions led to the proposed description of *Parcubacteria* as symbiontic/parasite organisms (Nelson and Stegen, 2015). Genes supporting an anaerobic lifestyle have also been detected, which appear to be involved in hydrogen and sulfur cycles in anoxic sediments (Elshahed et al., 2005; Wrighton et al., 2012). This would explain their presence in anoxic and sulfur rich sediments such as those of the Fuente de Piedra lagoon under study.

In the MS samples *Proteobacteria* was the most abundant phylum (61%), as reported for most coastal sediments (Zinger et al., 2011). The community was dominated by *Alphaproteobacteria* (34%) followed by *Gammaproteobacteria, Bacteroidetes, Deltaproteobacteria, Actinobacteria*, and *Planctomycetes*. *Gammaproteobacteria* and *Alphaproteobacteria* also dominated superficial marine sediments exposed to the Deepwater Horizon spill (Kostka et al., 2011). Under aerobic conditions, *Gammaproteobacteria* are generally associated to the early stages of aerobic hydrocarbon degradation, when alkanes and easily degradable hydrocarbons are degraded, while the abundance of *Alphaproteobacteria* increases in the later stages of degradation, when more recalcitrant hydrocarbon compounds such as PAHs predominate (Acosta-González et al., 2015; King et al., 2015). Therefore, the high proportion of *Alphaproteobacteria* in our sediment samples, which were a mixture of the oxic superficial layers and the deeper transition and anoxic zones, may be related to the aerobic hydrocarbon biodegradation stage in the community. The abundance of *Actinobacteria* might also be associated to later degradation stages, since they are involved in the degradation of both the long-chain alkane and aromatic fractions (Acosta-González et al., 2015; Acosta-González and Marqués, 2016). Unfortunately little is known about hydrocarbon degradation in anoxic conditions, which are the prevalent conditions in the deeper layers of the sediment, although members of the *Deltaproteobacteria* seem to play a relevant role in the process (Kimes et al., 2013; Kleindienst et al., 2014; Acosta-González and Marqués, 2016).

### General effect of PAHs on the bacterial community structure

**Figure 3:**
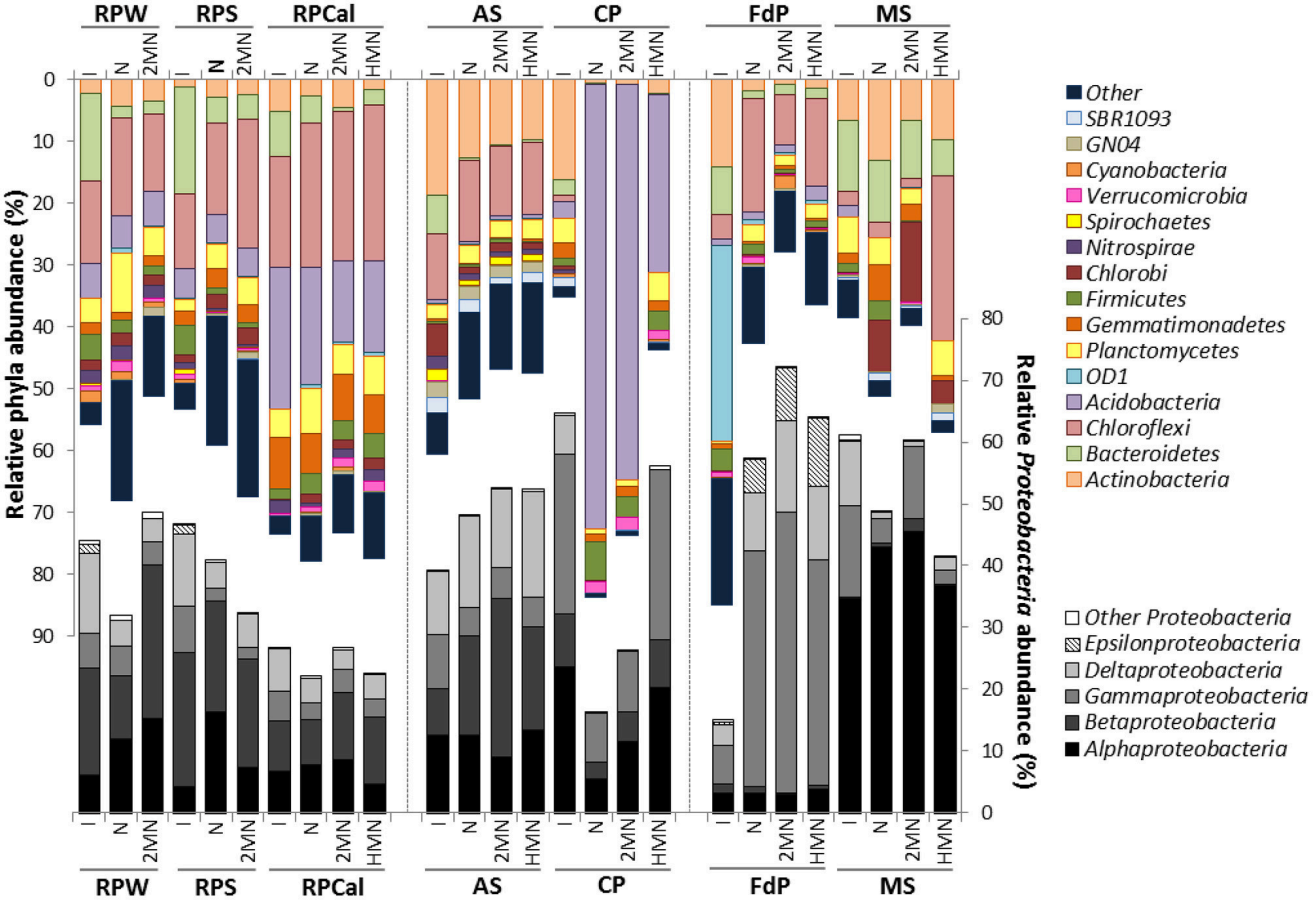
**Cumulative plot of bacterial phyla detected in the different enrichments**. The average values of the duplicate initial samples (Figure 2) are included for comparison, labeled as I. All other labels are as in Figure 2. Numerical values can be found in Table S5.

To evaluate the possible long-term effect of PAHs on the bacterial richness and diversity, we followed the changes in the community structure in the enrichment cultures (Figure 3) and analyzed the OTU richness and Chao diversity index in each case (Table 2). In the paddy soil enrichments we observed two different trends: Whilst the OTU number and Chao index in all the enrichments from Calasparra soil only showed a slight decrease, a significant reduction of the richness estimators in the Seville rice-paddy samples (RPS, RPW) was observed, although in this case the absence of the control culture with HMN hindered us from reaching a clear conclusion on the toxic effect of PAHs. No significant variations in OTU richness and Chao index were detected in the activated sludge samples, suggesting no overall adverse effect of naphthalene and 2MN on the community, as expected from a highly polluted environment. This contrasted with the toxic effect of PAHs on the nitrate reducing community inferred from the MPN results, and suggested that this type of metabolism constituted only a minor fraction of the population. It is worth noting that in the cultures used for the MPN enumeration, PAHs were provided as crystals and probably reached the saturating concentrations (i.e., 200 μm for naphthalene), whilst in the enrichments PAHs were supplied dissolved in HMN to keep concentrations below saturation (~70 μM, Ghoshal et al., 1996).

In contrast, the compost pile cultures were significantly affected by the culture conditions and especially by the presence of PAHs, which produced the lower values of richness estimators and species evenness. In the marine sediment samples the decrease in the number of OTUs and Chao index was significant in all cases, indicating that changes from the original sulfate-reducing metabolism to nitrate reduction conditions had a determinant detrimental effect on the community. In contrast, in the athalassohaline lagoon sediment samples the richness estimator increased in the three enrichments, suggesting a niche increment in the ecosystem as the samples switched from a sulfate-reducing environment to nitrate reducing conditions. The FdP lagoon is characterized by drastic changes in salinity throughout the year, depending on the local pluviometry, with variations ranging between 30 and 300 g/l in extremely dry years. Furthermore, the lagoon is surrounded by agricultural fields periodically supplemented with nitrogen fertilizers, which in the past drained into the lagoon. Interestingly, neither nitrate nor nitrite were detected in the FdP samples (Table 1), indicating a strong potential for denitrifying activity (Spence et al., 2005), which explains the positive response of the community to the change imposed on the respiratory regime.

**Figure 4:**
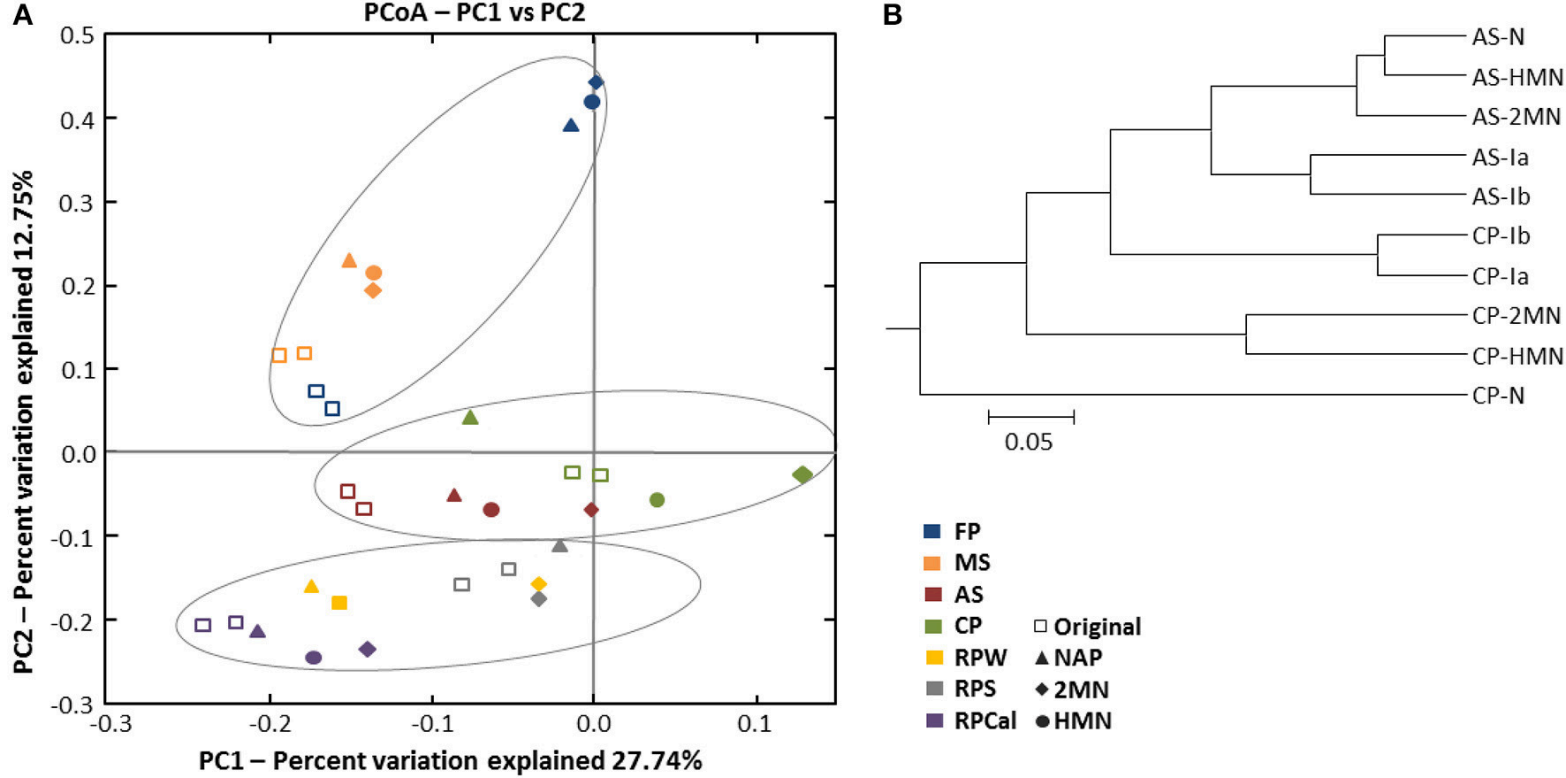
**(A)** Principal coordinate analysis (PCoA) of distances between OTUs present in the initial environmental samples and the enrichments obtained from them. The percentage of the variation between the samples by principal coordinates is indicated on the axes. **(B)** Unweighted pair group method with arithmetic average (UPGMA) cluster analysis of bacterial community structure in the compost pile (CP) and activated sludge (AS) environmental sample and enrichments from them using pyrosequencing analysis data based on Pearson correlation using OTUs (>97% sequence similarity) as the species data.

Principal coordinate analysis (PCoA) of the phylogenetic variation measured via UniFrac distances (Lozupone and Knight, 2005) revealed that an important community structuring factor was the original environment type where the samples were collected from (Figure 4A). The microbial communities, in fact, were clustered into three groups: Rice-paddy original samples and enrichments, activated sludge and compost pile original samples and enrichments, and, marine sediment and athalassohaline lagoon original samples and enrichments. To estimate if the structure of the community was affected by the addition of PAHs, we focused on the environment type subgroups. UPGMA, PCoA and significance analysis (two-sided Student's tests, Table S6) were performed using QIIME. Within the rice-paddy sample group the differences were minor, although statistically significant changes in RPS and RPW naphthalene and 2MN enrichments (*p* ≤ 0.05) were found as compared to the original sample. Naphthalene RPCal enrichment was also more affected than the HMN and 2MN cultures. No consistent differences between the HMN controls and the PAH enrichments were found in the activated sludge communities (*p* ≥ 0.05). Furthermore, the compost pile PAH enrichments were significantly distant from the initial sample and HMN control (*p* ≤ 0.05), especially the naphthalene culture, indicating an important phylogenetic variation of the community structure due to the presence of this PAH (Figure 4B, Table S6). Finally, in the MS and FdP samples no important differences could be detected between the control and the PAH-amended cultures, indicating that the community shift was mainly due to changes in the culture conditions (i.e., the shift to nitrate as terminal electron acceptor).

### Effect of PAHs on specific bacterial populations

**Figure 5:**
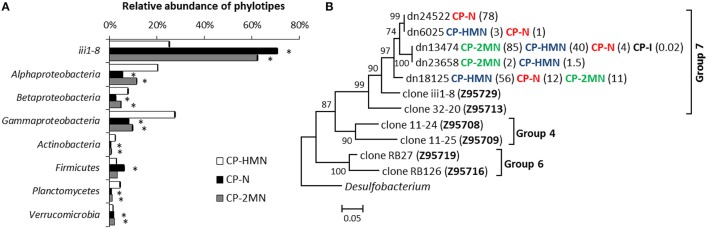
**Effect of PAHs on the relative abundance of the most relevant groups in the compost pile (CP) sample. (A)** Differences in the relative abundance of the most abundant groups between control (HMN), naphthalene (N), and 2-methyl-naphthalene (2MN) enrichments from the compost pile sample. Asterisks (^*^) indicate significant differences between rarefied samples according to the Mann-Whitney test (*p* < 0.01). **(B)** Evolutionary relationships of iii1-8 DS18 sequences retrieved from the compost pile initial sample and enrichments. The optimal Neighbour-Joining tree is shown. The bootstrap (1000 replicates) is shown next to the branches. The evolutionary distances are in the units of the number of base substitutions per site. Numbers in brackets indicate the relative abundance (%) of each sequence in the corresponding sample. The sequences of previously described *Acidobacteria* clones are included (Hugenholtz et al., 1998).

Over-imposed upon this general shift, we were able to detect some specific changes ascribable to the presence of PAHs. The compost pile was the sample where the presence of PAHs produced the major changes. In the naphthalene and 2MN enrichments we observed a decrease in the abundance of six phylotypes (*Proteobacteria, Actinobacteria, Bacteroidetes, Gemmatimonadetes, Planctomycetes, and SBR1093*). Specifically, *Alpha*- *Beta*- and *Gammaproteobacteria* decreased to a higher extent in naphthalene and 2MN treatments as compared to the HMN control. *Actinobacteria* also decreased significantly (from 16.2 to 1.3%) in all the enrichment cultures. In parallel an important increase of the relative abundance of the phyla *Acidobacteria, Firmicutes*, and *Verrucomicrobia* was observed. In particular *Acidobacteria* relative abundance increased in all the cultures, but the increase was extreme in naphthalene and 2MN enrichments (from 2.9 to 71.7 and 63.7%, respectively), where they became the dominant phylum (Figure 5A). The enriched *Acidobacteria* community was almost exclusively composed of the uncultured group iii1-8 (order DS18) (Figure 5B). Interestingly, a significant increase in this group was also observed in the PAH enrichments obtained from the rice-paddy samples (Table 3) where members of the order DS18 of the iii1-8 class, which were initially minor components of the community, increased up to 5.2% in the naphthalene enrichments from RPCal. The iii1-8 sequences retrieved from the different enrichments formed different clusters, and separated from the previously described iii1-8 sequences (Figure S2). Notably, the 5840 reads (78% of the sequences) from the CP naphthalene enrichments constituted a single OTU, distinct from the bulk of 2MN-enrichment sequences. Half of the sequences from the CP HMN control culture formed a distinct branch, whilst the other half belonged to the same OTU as the 2MN-enriched sequences. Furthermore, paddy soil PAH enriched sequences tended to cluster together, and separated from the CP PAH enriched sequences (Figure S2). *Acidobacteria* are among the most dominant phyla present in soils and are believed to play an important role in the ecology of this ecosystem. Unfortunately, the lack of acidobacterial isolates complicates the elucidation of their ecology and significance (Janssen, 2006; Jones et al., 2009). *Acidobacteria* class iii1-8 belongs to the monophyletic subdivisions 7 and was first identified in 1997 (Ludwig et al., 1997). Evidence suggests that pH is the best predictor of *Acidobacteria* abundance and distribution in soils, although the different subgroups are differentially affected by this variable: Some subgroups are favored at lower pH while some others, particularly sub-group 7, are positively correlated with higher pH values (Jones et al., 2009; Rousk et al., 2010). It is worth noting that in our experiments the pH remained neutral. In addition, a negative correlation with carbon availability and carbon mineralization rates has been observed, leading to the description of this group as slow-growing oligotrophs (Fierer et al., 2007). Furthermore, almost nothing is known about the role of *Acidobacteria* in aromatic degradation, and even less under anoxic conditions. Mukherjee et al. (2014) hypothesized that *Acidobacteria* phylum was not affected by hydrocarbon contamination levels and behaved as generalists in hydrocarbon polluted soils; however, a general increase of *Acidobacteria* in soil was observed in the presence of pyrene (Ren et al., 2015), and stable isotope probing of soils amended with ^13^C-labeled benzene identified a member of the *Acidobacteria* among the strains involved in benzene degradation (Xie et al., 2011). Although the function of *Acidobacteria* class iii1-8 in the communities analyzed here is difficult to predict, we could speculate that they are PAH resistant and may play a role in some step of the anaerobic degradation of PAHs, given their enrichment in PAH cultures under nitrate reducing conditions.

**Table 3:** **Relative abundance of ***Acidobacteria*** in the initial samples and enrichment cultures**.

**Sample**		**Taxon**
		**iii1-8; DS-18**
RPW	initial_A_	0.2
	initial_B_	0.69
	NAP	**1.05**
	2MN	**1.37**
RPS	initial_A_	0.76
	initial_B_	0.49
	NAP	**2.46**
	2MN	**2.07**
RPCal	initial_A_	1.43
	initial_B_	1.22
	NAP	**5.18**
	2MN	1.47
	HMN	1.68
CP	initial_A_	0
	initial_B_	0.02
	NAP	**70.55**
	2MN	**62.27**
	HMN	**25.2**

*Values showing differences with the initial sample are highlighted in bold*.

To a lesser extent but still interesting was the increment of the *Firmicutes* phylum, especially of members of the *Bacilli* class, which increased from 0.7 to 6% in the CP naphthalene enrichments but only to 3.3, and 3.0%, in the 2MN enrichment and HMN control, respectively. We observed the same trend in other enrichments, as in MS where *Bacilli* class increased from 1% in the original sample to 3% in the naphthalene enrichment, but decreased to 0.05 and 0.06% in the 2MN and HMN cultures, respectively. The same was true for the FdP samples where, despite a general decrease of the *Firmicutes* group, the *Bacilli* phylotypes increased especially in the naphthalene cultures. This increase of *Bacilli* relative abundance is noteworthy, since members of this class have been described as aerobic and anaerobic oil-degrading strains (Van Gestel et al., 2003; da Cruz et al., 2011; Maddela et al., 2016), which suggests a link between the increase of this group and the degradation process.

In the FdP enrichments we also observed a drastic change of the structure of the bacterial community in the enrichment cultures, although in this case the variation may be mainly attributed to the imposed culture conditions rather than to the presence of PAHs. The main changes detected were the great increase in members of the *Gammaproteobacteria, Deltaproteobacteria*, and *Epsilonprotebacteria* and the significant decrease of the candidate phylum *OD1*, which almost disappeared (from 31 to 0.33%). The order *Chromatiales* accounted for more than 70% of the increase in the *Gammaproteobacteria*. Members of this group have been shown to contain the gene complement for both sulfur oxidation and nitrate respiration, and the regulation of the two functions seems to be coordinated in these organisms (Baker et al., 2015). They are generally found in shallow sulfate-rich environments where they are thought to play a role in nitrate-dependent sulfur oxidation. The change from the original sulfate-reducing conditions in the athalassohaline lagoon to the nitrate reducing conditions in the FdP cultures may explain the enrichment of this phylogenetic group. Additionally, a general decrease of the other dominant phyla was observed, except for *Chloroflexi, Acidobacteria*, and *Planctomycetes*. The increase in members of the *Anaerolineae* of the *Chloroflexi* and of *Planctomycetes* was especially noticeable in the naphthalene enrichments. The increase of *Anaerolineae*, a group which has been retrieved from many natural and artificial ecosystems, among which hypersaline lakes, agriculture soils, waste water treatment plants and microbial fuel cells, is probably not related to nitrate respiration, as all cultured strains of this class do not utilize nitrate as terminal electron acceptor (Yamada et al., 2006). Rather it seems that these organisms, with a fermentative anaerobic metabolism, are using carbohydrate and cells remains produced in the enrichment cultures (Yamada and Sekiguchi, 2009).

In the enrichments from marine sediment, the change from the initial sulfate respiration regime to the imposed nitrate reduction metabolism produced less drastic changes in the community structure than expected. Although the changes in the three enrichments were parallel, differences between the HMN control and the PAH amended enrichments were noticeable. *Alphaproteobacteria* remained the dominant class among the *Proteobacteria* (34.53–45.46%), increasing in all three conditions, although to a higher extent in the naphthalene and 2-MN cultures. Interestingly a higher increase in the relative abundance of *Actinobacteria* was observed in the naphthalene enrichments. Furthermore, the phylotypes *Gemmatimonadetes* and *Firmicutes* increased in the PAH enrichments and decreased in the HMN control cultures (Figure 3, Table S5). Altogether, these changes in the community may reflect a connection with resistance to PAHs. The decrease of the phyla *Bacteroidetes, Planctomycetes, Acidobacteria*, and *WS3* were similar in all the culture conditions, and thus could not be attributed to the presence of PAHs.

Changes in the remaining enrichments were modest. Incubation of rice-paddy samples with PAHs under nitrate reducing conditions produced only slight changes in the bacterial communities, and most of them were attributable to the new culture medium, since similar shifts were observed in the presence and absence of PAHs. Nevertheless, it is important to point out that communities with a high diversity, such as those present in soil, appear to be more stable when under stress (McCann, 2000). Looking in more details at some of the specific changes, we observed that *Bacteroidetes* relative abundance decreased significantly in the naphthalene and 2MN enrichments, suggesting sensitivity to the presence of PAHs. Although this phylum is widespread in both pristine and contaminated environments, a similar decrease of *Bacteroidetes* abundance has been recently reported in soil microcosms contaminated with PAHs (Sawulski et al., 2014). *Firmicutes*, especially members of the *Clostridia*, increased in the presence of naphthalene and 2MN enrichments whilst the changes in the relative abundance of *Bacilli* did not show a clear pattern. Several anaerobic hydrocarbon degraders belonging to the *Clostridia* have been isolated (Morasch et al., 2004), or identified as hydrocarbon degraders in the environment (Kunapuli et al., 2007; Fowler et al., 2012), although most of them are SRB, iron reducers and methanogens. The relative abundance of *Acidobacteria* remained unchanged or slightly reduced in the difference paddy soil enrichments and controls. However, as discussed above, an interesting enrichment of the *Acidobacteria* class iii1-8 was observed in all the samples with N and 2-MN (Table 3).

Finally, a general increase of the *Proteobacteria* relative abundance was observed in AS enrichments, although the final proportion of the different classes was different in the three culture conditions (Figure 3). The *Betaproteobateria* relative abundance increased in all cultures but especially in the 2MN enrichment, reaching 25% of the community. The remaining phyla only showed slight changes in the cultures with respect to their relative abundance in the initial sample, and no clear effect of the presence of PAHs was evidenced. Overall no clear association could be established between the observed changes in the community structure and the presence of PAHs.

### Isolation of nitrate reducing bacteria from PAH enrichments

The growth of the different cultures was checked by following the respiratory activity as nitrate consumption. Nitrate respiration was observed in some enrichment cultures on PAHs (data not shown). However, significant differences between the control HMN cultures and the PAH amended cultures were only observed in the CP culture with 2MN as the carbon source. The nitrate consumed after the last transfer was below 3 mM, which would correspond to a maximum consumption of 310 μM of 2MN if full denitrification of nitrate was assumed (Mihelcic and Luthy, 1988). The concentration of PAHs used in the HMN solution was too high to allow accurate determination of PAH consumption at these levels. We therefore attempted to isolate strains able to grow on solid media under denitrifying conditions with naphthalene or 2MN as carbon source. Several strains able to consistently grow with naphthalene as carbon source could be isolated both on agar plates prepared under anoxic conditions and using the agar shakes method (Table S7). These included species of the *Bacillus* and *Pseudomonas* genera, among others. However, the growth phenotype could not be reproduced in liquid medium, where no nitrate respiration was observed in the presence of naphthalene after several months. A possible reason for this discrepancy might be that despite anaerobic preparation and incubation of the solid media cultures, oxygen could be a contaminant and allow microaerophylic growth of the strains. However, no aerobic growth of the isolates in liquid medium was obtained either with the corresponding PAHs as carbon source. Interestingly, strains of *P. stutzeri* were isolated with both methods, which were also the strain originally described by Röckne et al. (2000) as naphthalene degraders. Although some strains of this species are known aerobic naphthalene degraders (Lauber et al., 2009), the strains isolated in this study were unable to grow aerobically with naphthalene as carbon source. It is worth noting that in both isolation methods, no growth on solid medium was observed when naphthalene was omitted from the medium.

### Aromatic anaerobic degradation genes

In SRB naphthalene and 2MN degradation pathways have been elucidated and primers for the amplification of the fumarate adding enzyme naphthyl-2-methylsuccinate synthase gene (*nmsA*) have been used to detect the presence of the pathway in environmental samples (Winderl et al., 2007; Acosta-González et al., 2013b; von Netzer et al., 2013). To evaluate the anaerobic hydrocarbon degrading potential in the environmental samples and enrichments we tested different previously designed primer pairs to detect *nmsA* and *bssA*, coding for the homologous toluene pathway degradation enzyme benzylsuccinate synthase. In addition, we used primers for the amplification of naftoil-CoA reductase (*ncr*), the central step in naphthalene degradation (Morris et al., 2014). Only the primer set for the detection of fumarate addition enzymes consistently rendered PCR amplification products. In total, twelve clone libraries were constructed from DNA isolated from the samples giving PCR amplification products, and sixteen clones from each library were sequenced. Finally we were able to retrieve *bssA* homologs from rice-paddy initial samples (RPW and RPS) and enrichments (RPS-N, RPCal-N, RPCal-2MN and RPCal-HMN), from the AS initial sample and naphthalene and 2-MN enrichments and from the naphthalene enrichment of the marine sediment (Figure S3). The resulting amplified *bssA*-like sequences were translated into amino acid sequences and compared with available sequences in the databases to build a phylogenetic tree (Figure S4). All the sequences clustered within the cluster I of toluene-specific BssA sequences (*bssA sensu stricto*), which included sequences from well-characterized toluene-degrading strains and from environmental samples such as hydrocarbon-polluted aquifers, aquifer sediments, oil fields, sludge, and polluted soils. Interestingly, within cluster I the sequences retrieved from marine sediments and activated sludge formed a distinct cluster, which was already observed for sequences of marine origin (Acosta-González et al., 2013b). These results reflect the absence of efficient “universal” primers for *nms* and *ncr* genes, considering the limited number of anaerobic PAH degradation gene sequences available (DiDonato et al., 2010; Selesi et al., 2010). Moreover, the reference genes used to design the probes were obtained from sulfate-reducing organisms; nitrate dependent PAH degradation may follow a different pathway.

## Concluding remarks

In this study we were able to detect in some environments the presence of a significant nitrate reducing bacteria community able to thrive using PAHs as carbon source. Enrichment cultures under anoxic conditions with nitrate as terminal electron acceptor and naphthalene or 2MN as the carbon source produced shifts in the structure of the community connected to the change imposed on the respiratory regime, from aerobic, microaerophilic, and sulfate reducing conditions to nitrate respiration. However, some changes specifically related to the presence of PAHs could be identified. The relative abundance of *Bacteroidetes* and *Actinobacteria* was reduced to different extents in PAH enrichments, which was attributed to the sensitivity of these groups to the toxicity of PAHs. In contrast, some groups increased significantly. Especially noticeable was the selection for the uncultured *Acidobacteria* class iii1-8, particularly. in the compost-pile sample, where the iii1-8 community increased under all culture conditions, but to a significantly higher extent in the naphthalene cultures, and was also clearly observed in all those naphthalene enrichments where this group was present in the initial samples. Unfortunately, a direct connection between enrichment in iii1-8 sequences and PAH degradation under nitrate reducing conditions could not be established, and the ecological role of this group in the enrichments is unclear. Members of the *Bacilli* were also enriched in most PAHs cultures, and strains belonging to this group could be isolated on solid media with naphthalene as carbon source. However, these isolates were unable to grow with naphthalene in liquid media. The failure of our attempts to isolate nitrate reducing PAH-degrading bacteria may suggest that in the environment, PAH degradation is a complex process in which different organisms are involved. Based on the experience of the past decades in the field of biodegradation, both under aerobic and anoxic conditions, this inability to identify and isolate NRBs able to degrade common natural compounds such as the PAHs used here is unexpected, especially considering that NRBs capable of degrading benzene (an unsubstituted monoaromatic with a structure similar to naphthalene) have been identified (Chakraborty et al., 2005; Kasai et al., 2006). However, in this latter case an aerobic metabolism based on internally produce oxygen through the potential dismutation of the nitric oxide produced during respiration has been proposed (Meckenstock et al., 2016). NRBs are generally facultative anaerobes, with the capacity to carry out a mixed oxic/anoxic type of metabolism (Valderrama et al., 2012). It would thus be expected that in some environments processes as complex as PAH degradation would require the coordinated activity of both types of metabolisms. The strict anaerobic conditions imposed in our enrichments would prevent the development of such consortia.

## Author contributions

The study was conceived and designed by SM and SMM. SMM, SM, and DP conducted environmental sampling, SMM, PM, DP, and PB performed all laboratory analyses, SMM performed all computational analyses, and SM and SMM wrote the paper.

## Funding

This work was supported by the European Regional Development Fund FEDER and grants from the Junta de Andalucía (P08-CVI03591), from the Spanish Ministry of Economy and Competitiveness (BIO2014-54361-R) and European Union's 7th Framework Program under Grant Agreement 312139. SMM was the recipient of a fellowship within the CSIC JAE PREDOC program. DP was the recipient of a Junta de Andalucía Predoctoral Fellowship.

### Conflict of interest statement

The authors declare that the research was conducted in the absence of any commercial or financial relationships that could be construed as a potential conflict of interest.

## Supplementary material

Table S1 **(A)** Bacterial universal primers 6F and 532R containing 5′ tags with multiplex identifier (MID) and sequencing adapters used in this study for the initial samples. **(B)** Bacterial universal primers 6F and 532R containing 5′ tags with multiplex identifier (MID) and sequencing adapters used in this study for the enrichment culture samples.

Table S2 **Functional-gene primer sequences used in this study**.

Table S3 **(A)** Aliphatic hydrocarbon composition and abundance in the original samples. **(B)** PAH composition and abundance in the original samples.

Table S4 **Most probable number counts of nitrate reducing bacteria in the environmental samples**. Carbon source are naphthalene (NAP), 2-methylnaphthalene (2MN), 2-naphtoic acid (2NA), anthracene (ANT), acetate (Ace), and no added carbon source (na).

Table S5 **Relative abundance of the different phylotypes in the samples analyzed in this work at the genus level**.

Table S6 **Significance tests performed using a two-sided Student's two-sample ***t***-test comparing the Unifrac weighted distance**.

Table S7 **Taxonomic affiliation of strains isolated on solid medium with PAHs as carbon source**.

Figure S1 **Rarefaction curves based on bacterial OTUs at a dissimilarity level of 3% of the initial samples and enrichments as calculated by Qiime**.

Figure S2 **Evolutionary relationships of iii1-8 DS18 sequences retrieved from the rice-paddy and compost-pile initial sample and enrichments**. The optimal Neighbour-Joining tree is shown. The bootstrap (1000 replicates) is shown next to the branches. The evolutionary distances are in the units of the number of base substitutions per site. Numbers in brackets are the percentage of each sequence type over the complete set of iii1-8 reads in each sample. The sequences of three different *Acidobacteria* subgroups are included (Hugenholtz et al., 1998). Numbers in brackets indicate the relative abundance (%) of each sequence in the corresponding sample.

Figure S3 **Frequency distribution of ***bssA***-like and non-***bssA***-like sequence types in the different libraries constructed from the samples**.

Figure S4 **Phylogeny of partial BssA-like amino acid sequences retrieved from initial samples and enrichments**. Initial samples were rice-paddy water (RPW-I; gray), rice-paddy soil (RPS-I; blue), and activated sludge (AS-I; red). Enrichments were rice-paddy soil naphthalene (RPS-N; blue), rice-paddy soil Calasparra naphthalene (RPCal-N; black), rice-paddy soil Calasparra 2MN (RPCal-2MN; black), rice-paddy soil Calasparra HMN (RPCal-HMN; black), activated sludge naphthalene (AS-N; red), activated sludge 2MN (AS-2MN; red), and marine sediments naphthalene (MS-N; green). The closest relatives, detected in anaerobic hydrocarbon-oxidizing strains or enrichments, and putative environmental sequences available in the databases at the beginning of this study are included. Cluster I includes the canonical, toluene-specific BssA sequences; cluster II includes sequences associated with the oxidation of BTEX compounds; cluster III includes the 2-methyl-naphthalene-specific *nmsA* sequences, putative *bssA* gene from the non-proteobacterial *Desulfotomaculum* sp. strain Ox39 and sequences retrieved from enrichments able to grow on o-xylene as the only carbon source; cluster IV comprises *assA* sequences. Codes in brackets are the protein GenBank accession numbers. Numbers in square brackets are the number of sequences found in the corresponding library. The tree was rooted with pyruvate formate lyase paralogs as an out-group.
