# Supplementary material for: Polycyclic Aromatic Hydrocarbon-Induced Changes in Bacterial Community Structure under Anoxic Nitrate Reducing Conditions
Source: Front Microbiol. 2016 Nov 8;7:1775. doi: 10.3389/fmicb.2016.01775 (PMC5099901; doi:10.3389/fmicb.2016.01775)
Supplement: Table S1 — (A) Bacterial universal primers 6F and 532R containing 5′ tags with multiplex identifier (MID) and sequencing adapters used in this study for the initial samples. (B) Bacterial universal primers 6F and 532R containing 5′ tags with multiplex identifier (MID) and sequencing adapters used in this study for the enrichment culture samples. [file Table1.DOCX]

***Supplementary material***

**Polycyclic aromatic hydrocarbon-induced changes in bacterial community structure under anoxic nitrate reducing condition.**

Sophie-Marie Martirani-Von Abercron, Daniel Daniel, Patricia Benito-Santano, Patricia Marín and Silvia Marqués^*^

Estación Experimental del Zaidín, Department of Environmental Protection, Consejo Superior de Investigaciones Científicas, Granada, Spain.

*Author for correspondence: Silvia Marqués, Estación Experimental del Zaidín, CSIC, C/. Profesor Albareda nº1, E-18008 Granada, Spain, [silvia@eez.csic.es](mailto:silvia@eez.csic.es)

**Table S1a.** Bacterial universal primers 6F and 532R containing 5′ tags with multiplex identifier (MID) and sequencing adapters used in this study for the initial samples.

| **Sample name** | **Primer name** | **Adaptor** | **Key** | **MID** | **Primer** | **Primer position** |
| --- | --- | --- | --- | --- | --- | --- |
| **RPW-I_a_** | M3A | CGTATCGCCTCCCTCGCGCCA | TCAG | AGACGCACTC | TCAGAGTTTGATCCTGGCTCAG | 6F |
|  | M3B | CTATGCGCCTTGCCAGCCCGC | TCAG | AGACGCACTC | CACCGCGGCKGCTGGCAC | 532R |
| **RPW- I_b_** | M4A | CGTATCGCCTCCCTCGCGCCA | TCAG | AGCACTGTAG | TCAGAGTTTGATCCTGGCTCAG | 6F |
|  | M4B | CTATGCGCCTTGCCAGCCCGC | TCAG | AGCACTGTAG | CACCGCGGCKGCTGGCAC | 532R |
| **RPS- I_a_** | M5A | CGTATCGCCTCCCTCGCGCCA | TCAG | ATCAGACACG | TCAGAGTTTGATCCTGGCTCAG | 6F |
|  | M5B | CTATGCGCCTTGCCAGCCCGC | TCAG | ATCAGACACG | CACCGCGGCKGCTGGCAC | 532R |
| **RPS- I_b_** | M6A | CGTATCGCCTCCCTCGCGCCA | TCAG | ATATCGCGAG | TCAGAGTTTGATCCTGGCTCAG | 6F |
|  | M6B | CTATGCGCCTTGCCAGCCCGC | TCAG | ATATCGCGAG | CACCGCGGCKGCTGGCAC | 532R |
| **RPCal- I_a_** | M7A | CGTATCGCCTCCCTCGCGCCA | TCAG | CGTGTCTCTA | TCAGAGTTTGATCCTGGCTCAG | 6F |
|  | M7B | CTATGCGCCTTGCCAGCCCGC | TCAG | CGTGTCTCTA | CACCGCGGCKGCTGGCAC | 532R |
| **RPCal- I_b_** | M8A | CGTATCGCCTCCCTCGCGCCA | TCAG | CTCGCGTGTC | TCAGAGTTTGATCCTGGCTCAG | 6F |
|  | M8B | CTATGCGCCTTGCCAGCCCGC | TCAG | CTCGCGTGTC | CACCGCGGCKGCTGGCAC | 532R |
| **AS- I_a_** | M9A | CGTATCGCCTCCCTCGCGCCA | TCAG | TAGTATCAGC | TCAGAGTTTGATCCTGGCTCAG | 6F |
|  | M9B | CTATGCGCCTTGCCAGCCCGC | TCAG | TAGTATCAGC | CACCGCGGCKGCTGGCAC | 532R |
| **AS- I_b_** | M10A | CGTATCGCCTCCCTCGCGCCA | TCAG | TCTCTATGCG | TCAGAGTTTGATCCTGGCTCAG | 6F |
|  | M10B | CTATGCGCCTTGCCAGCCCGC | TCAG | TCTCTATGCG | CACCGCGGCKGCTGGCAC | 532R |
| **CP- I_a_** | M11A | CGTATCGCCTCCCTCGCGCCA | TCAG | TGATACGTCT | TCAGAGTTTGATCCTGGCTCAG | 6F |
|  | M11B | CTATGCGCCTTGCCAGCCCGC | TCAG | TGATACGTCT | CACCGCGGCKGCTGGCAC | 532R |
| **CP- I_b_^1^** | M12A | CGTATCGCCTCCCTCGCGCCA | TCAG | TACTGAGCTA | TCAGAGTTTGATCCTGGCTCAG | 6F |
|  | M12A | CTATGCGCCTTGCCAGCCCGC | TCAG | TACTGAGCTA | CACCGCGGCKGCTGGCAC | 532R |
| **FdP- I_a_** | RL90A | CGTATCGCCTCCCTCGCGCCA | TCAG | CGCGCTATACT | TCAGAGTTTGATCCTGGCTCAG | 6F |
|  | RL90B | CTATGCGCCTTGCCAGCCCGC | TCAG | CGCGCTATACT | CACCGCGGCKGCTGGCAC | 532R |
| **FdP- I_b_^1^** | M12A | CGTATCGCCTCCCTCGCGCCA | TCAG | TACTGAGCTA | TCAGAGTTTGATCCTGGCTCAG | 6F |
|  | M12A | CTATGCGCCTTGCCAGCCCGC | TCAG | TACTGAGCTA | CACCGCGGCKGCTGGCAC | 532R |
| **MS- I_a_** | RL84A | CGTATCGCCTCCCTCGCGCCA | TCAG | CGAGACACTAT | TCAGAGTTTGATCCTGGCTCAG | 6F |
|  | RL84B | CTATGCGCCTTGCCAGCCCGC | TCAG | CGAGACACTAT | CACCGCGGCKGCTGGCAC | 532R |
| **MS- I_b_** | RL85A | CGTATCGCCTCCCTCGCGCCA | TCAG | CGAGAGTGTGT | TCAGAGTTTGATCCTGGCTCAG | 6F |
|  | RL85B | CTATGCGCCTTGCCAGCCCGC | TCAG | CGAGAGTGTGT | CACCGCGGCKGCTGGCAC | 532R |

^1^The samples analyzed with identical MID primers were sequenced in different runs.

**Table S1b.** Bacterial universal primers 6F and 532R containing 5′ tags with multiplex identifier (MID) and sequencing adapters used in this study for the enrichment culture samples.

| **Sample name** | **Primer name** | **Adaptor** | **Key** | **MID** | **Primer** | **Primer position** |
| --- | --- | --- | --- | --- | --- | --- |
| **RPW-N^1^** | M3A | CGTATCGCCTCCCTCGCGCCA | TCAG | AGACGCACTC | TCAGAGTTTGATCCTGGCTCAG | 6F |
|  | M3B | CTATGCGCCTTGCCAGCCCGC | TCAG | AGACGCACTC | CACCGCGGCKGCTGGCAC | 532R |
| **RPW-2MN^1^** | M5A | CGTATCGCCTCCCTCGCGCCA | TCAG | ATCAGACACG | TCAGAGTTTGATCCTGGCTCAG | 6F |
|  | M5B | CTATGCGCCTTGCCAGCCCGC | TCAG | ATCAGACACG | CACCGCGGCKGCTGGCAC | 532R |
| **RPS-N^1^** | M5A | CGTATCGCCTCCCTCGCGCCA | TCAG | ATCAGACACG | TCAGAGTTTGATCCTGGCTCAG | 6F |
|  | M5B | CTATGCGCCTTGCCAGCCCGC | TCAG | ATCAGACACG | CACCGCGGCKGCTGGCAC | 532R |
| **RPS-2MN** | M6A | CGTATCGCCTCCCTCGCGCCA | TCAG | ATATCGCGAG | TCAGAGTTTGATCCTGGCTCAG | 6F |
|  | M6B | CTATGCGCCTTGCCAGCCCGC | TCAG | ATATCGCGAG | CACCGCGGCKGCTGGCAC | 532R |
| **RPCal-N** | M7A | CGTATCGCCTCCCTCGCGCCA | TCAG | CGTGTCTCTA | TCAGAGTTTGATCCTGGCTCAG | 6F |
|  | M7B | CTATGCGCCTTGCCAGCCCGC | TCAG | CGTGTCTCTA | CACCGCGGCKGCTGGCAC | 532R |
| **RPCal-2MN** | RL89A | CGTATCGCCTCCCTCGCGCCA | TCAG | CGCGATCGTAT | TCAGAGTTTGATCCTGGCTCAG | 6F |
|  | RL89B | CTATGCGCCTTGCCAGCCCGC | TCAG | CGCGATCGTAT | CACCGCGGCKGCTGGCAC | 532R |
| **RPCal-HMN** | M8A | CGTATCGCCTCCCTCGCGCCA | TCAG | CTCGCGTGTC | TCAGAGTTTGATCCTGGCTCAG | 6F |
|  | M8B | CTATGCGCCTTGCCAGCCCGC | TCAG | CTCGCGTGTC | CACCGCGGCKGCTGGCAC | 532R |
| **AS-N** | M9A | CGTATCGCCTCCCTCGCGCCA | TCAG | TAGTATCAGC | TCAGAGTTTGATCCTGGCTCAG | 6F |
|  | M9B | CTATGCGCCTTGCCAGCCCGC | TCAG | TAGTATCAGC | CACCGCGGCKGCTGGCAC | 532R |
| **AS-2MN^1^** | 128A | CGTATCGCCTCCCTCGCGCCA | TCAG | CACTCGCACG | TCAGAGTTTGATCCTGGCTCAG | 6F |
|  | 128B | CTATGCGCCTTGCCAGCCCGC | TCAG | CACTCGCACG | CACCGCGGCKGCTGGCAC | 532R |
| **AS-HMN** | M10A | CGTATCGCCTCCCTCGCGCCA | TCAG | TCTCTATGCG | TCAGAGTTTGATCCTGGCTCAG | 6F |
|  | M10B | CTATGCGCCTTGCCAGCCCGC | TCAG | TCTCTATGCG | CACCGCGGCKGCTGGCAC | 532R |
| **FdP-N^1^** | M3A | CGTATCGCCTCCCTCGCGCCA | TCAG | AGACGCACTC | TCAGAGTTTGATCCTGGCTCAG | 6F |
|  | M3B | CTATGCGCCTTGCCAGCCCGC | TCAG | AGACGCACTC | CACCGCGGCKGCTGGCAC | 532R |
| **FdP-2MN** | RL93A | CGTATCGCCTCCCTCGCGCCA | TCAG | CGTATAGTGCT | TCAGAGTTTGATCCTGGCTCAG | 6F |
|  | RL93B | CTATGCGCCTTGCCAGCCCGC | TCAG | CGTATAGTGCT | CACCGCGGCKGCTGGCAC | 532R |
| **FdP-HMN** | 131A | CGTATCGCCTCCCTCGCGCCA | TCAG | CGACAGCGAG | TCAGAGTTTGATCCTGGCTCAG | 6F |
|  | 131B | CTATGCGCCTTGCCAGCCCGC | TCAG | CGACAGCGAG | CACCGCGGCKGCTGGCAC | 532R |
| **CP-N** | M11A | CGTATCGCCTCCCTCGCGCCA | TCAG | TGATACGTCT | TCAGAGTTTGATCCTGGCTCAG | 6F |
|  | M11B | CTATGCGCCTTGCCAGCCCGC | TCAG | TGATACGTCT | CACCGCGGCKGCTGGCAC | 532R |
| **CP-2MN** | M4A | CGTATCGCCTCCCTCGCGCCA | TCAG | AGCACTGTAG | TCAGAGTTTGATCCTGGCTCAG | 6F |
|  | M4B | CTATGCGCCTTGCCAGCCCGC | TCAG | AGCACTGTAG | CACCGCGGCKGCTGGCAC | 532R |
| **CP-HMN** | M12A | CGTATCGCCTCCCTCGCGCCA | TCAG | TACTGAGCTA | TCAGAGTTTGATCCTGGCTCAG | 6F |
|  | M12A | CTATGCGCCTTGCCAGCCCGC | TCAG | TACTGAGCTA | CACCGCGGCKGCTGGCAC | 532R |
| **MS-N** | RL84A | CGTATCGCCTCCCTCGCGCCA | TCAG | CGAGACACTAT | TCAGAGTTTGATCCTGGCTCAG | 6F |
|  | RL84B | CTATGCGCCTTGCCAGCCCGC | TCAG | CGAGACACTAT | CACCGCGGCKGCTGGCAC | 532R |
| **MS-2MN^1^** | 128A | CGTATCGCCTCCCTCGCGCCA | TCAG | CACTCGCACG | TCAGAGTTTGATCCTGGCTCAG | 6F |
|  | 128B | CTATGCGCCTTGCCAGCCCGC | TCAG | CACTCGCACG | CACCGCGGCKGCTGGCAC | 532R |
| **MS-HMN** | RL85A | CGTATCGCCTCCCTCGCGCCA | TCAG | CGAGAGTGTGT | TCAGAGTTTGATCCTGGCTCAG | 6F |
|  | RL85B | CTATGCGCCTTGCCAGCCCGC | TCAG | CGAGAGTGTGT | CACCGCGGCKGCTGGCAC | 532R |

^1^The samples analyzed with identical MID primers were sequenced in different runs.
