# Supplementary material for: Polycyclic Aromatic Hydrocarbon-Induced Changes in Bacterial Community Structure under Anoxic Nitrate Reducing Conditions
Source: Front Microbiol. 2016 Nov 8;7:1775. doi: 10.3389/fmicb.2016.01775 (PMC5099901; doi:10.3389/fmicb.2016.01775)
Supplement: Table S2 — Functional-gene primer sequences used in this study. [file Table2.DOCX]

***Supplementary material***

**Polycyclic aromatic hydrocarbon-induced changes in bacterial community structure under anoxic nitrate reducing condition.**

Sophie-Marie Martirani-Von Abercron, Daniel Daniel, Patricia Benito-Santano, Patricia Marín and Silvia Marqués^*^

Estación Experimental del Zaidín, Department of Environmental Protection, Consejo Superior de Investigaciones Científicas, Granada, Spain.

*Author for correspondence: Silvia Marqués, Estación Experimental del Zaidín, CSIC, C/. Profesor Albareda nº1, E-18008 Granada, Spain, [silvia@eez.csic.es](mailto:silvia@eez.csic.es)

**Table S2.** Functional gene primer sequences used in this study.

| **Target gene** | **Primer set** | **5′-3′ sequence** | **Reference** |
| --- | --- | --- | --- |
| *bssA* | 7772f  8546r | GACATGACCGACGCSATYCT  TCGTCGTCRTTGCCCCAYTT | Winderl et al., 2007 |
| *bssA, nmsA* | 7768f  8543r | CAAYGATTTAACCRACGCCAT  TCGTCRTTGCCCCAYTTNGG | Von Netzer et al., 2013 |
| *nmsA* | 7363f  7374f  8543r | TCGCCGAGAATTTCGAYTTG  TTCGAYTTGAGCGACAGCGT  TCGTCRTTGCCCCAYTTNGG | Von Netzer et al., 2013 |
| *ncr* | Ncr1f  Ncr1r | CGTTATWCKCCYTGCCGTG  CGATAAGCCATRCADATRGG | Morris et al., 2014 |
| *ncr* | Ncr2f  Ncr2r | TGGACAAAYAAAMGYACVGAT  GATTCCGGCTTTTTTCCAAVT | Morris et al., 2014 |
