# Supplementary material for: Polycyclic Aromatic Hydrocarbon-Induced Changes in Bacterial Community Structure under Anoxic Nitrate Reducing Conditions
Source: Front Microbiol. 2016 Nov 8;7:1775. doi: 10.3389/fmicb.2016.01775 (PMC5099901; doi:10.3389/fmicb.2016.01775)
Supplement: Table S3 — (A) Aliphatic hydrocarbon composition and abundance in the original samples. (B) PAH composition and abundance in the original samples. [file Table3.DOCX]

***Supplementary material***

**Polycyclic aromatic hydrocarbon-induced changes in bacterial community structure under anoxic nitrate reducing condition.**

Sophie-Marie Martirani-Von Abercron, Daniel Daniel, Patricia Benito-Santano, Patricia Marín and Silvia Marqués^*^

Estación Experimental del Zaidín, Department of Environmental Protection, Consejo Superior de Investigaciones Científicas, Granada, Spain.

*Author for correspondence: Silvia Marqués, Estación Experimental del Zaidín, CSIC, C/. Profesor Albareda nº1, E-18008 Granada, Spain, [silvia@eez.csic.es](mailto:silvia@eez.csic.es)

**Table S3a.** Aliphatic hydrocarbon composition and abundance in the initial samples.

| **Compound** | **RPW** | **RPS** | **RPCal** | **AS** | **CP** | **FdP** | **MS** |
| --- | --- | --- | --- | --- | --- | --- | --- |
| **nC9** | nd | nd | nd | 0.17±0.01 | 0.17±0.02 | 0.24±0.04 | 0.12±0.09 |
| **nC10** | nd | nd | nd | 2.92±0.36 | 0.04±0.006 | 0.08±0.04 | 0.04±0.01 |
| **nC11** | 0.16±0.01 | 0.15±0.03 | 0.193±0.007 | 0.42±0.003 | 0.08±0.01 | 0.24±0.02 | 0.10±0.07 |
| **nC12** | 0.16±0.02 | 0.16±0.02 | 0.129±0.04 | 12.97±1.26 | 0.05±0.01 | 0.18±0.03 | 0.05±0.02 |
| **nC13** | nd | nd | nd | 3.04±0.36 | 0.04±0.07 | nd | 0.04±0.01 |
| **nC14** | 0.54±0.15 | 0.48±0.08 | 0.41±0.08 | 14.13±2.24 | 0.11±0.01 | 0.54±0.02 | 0.17±0.09 |
| **nC15** | 0.11±0.02 | 0.12±0.02 | 0.11±0.05 | nd | 0.17±0.04 | 0.19±0.02 | 0.04±0.01 |
| **nC16** | 0.62±0.32 | 0.53±0.06 | 0.481±0.001 | 7.12±2.66 | 0.17±0.03 | 0.76±0.10 | 0.24±0.12 |
| **nC17** | 0.26±0.03 | 0.30±0.04 | 0.39±0.16 | nd | 0.40±0.22 | 0.46±0.04 | 0.12±0.06 |
| **nC18** | 0.74±0.30 | 0.72±0.09 | 0.50±0.03 | 2.93±1.35 | 0.51±0.03 | 0.92±0.16 | 0.29±0.17 |
| **nC19** | 0.09±0.00 | 0.10±0.02 | 0.06±0.01 | nd | nd | 0.09±0.004 | 0.02±0.01 |
| **nC20** | 0.68±0.24 | 0.68±0.09 | 0.433±0.001 | 1.45±0.37 | nd | 0.85±0.11 | 0.27±0.17 |
| **nC21** | nd | nd | 0.03±0.23 | nd | nd | 0.13±0.14 | nd |
| **nC22** | 0.46±0.17 | 0.47±0.09 | 0.27±0.01 | nd | nd | 0.79±0.40 | 0.18±0.19 |
| **nC23** | 0.02±0.001 | 0.02±0.001 | 0.006±0.001 | nd | nd | 0.70±1.18 | nd |
| **nC24** | 0.15±0.12 | 0.17±0.05 | <dl | nd | nd | 1.74±2.72 | 0.08±0.08 |
| **nC25** | nd | <dl | <dl | nd | nd | 2.44±4.65 | nd |
| **nC26** | <dl | <dl | <dl | nd | nd | 3.32±6.15 | <dl |
| **nC27** | nd | nd | <dl | nd | nd | 3.67±6.99 | nd |
| **nC28** | <dl | nd | nd | nd | nd | 3.34±6.37 | nd |
| **nC29** | nd | nd | <dl | nd | nd | 2.52±4.94 | nd |
| **nC30** | nd | nd | nd | nd | nd | 1.69±3.31 | nd |
| **nC31** | nd | nd | nd | nd | nd | 1.01±1.98 | nd |
| **nC32** | nd | nd | nd | nd | nd | 0.43±0.85 | nd |
| **nC33** | nd | nd | nd | nd | nd | nd | nd |
| **nC34** | nd | nd | nd | nd | nd | nd | nd |
| **nC35** | nd | nd | nd | nd | nd | nd | nd |
| **nC36** | nd | nd | nd | nd | nd | nd | nd |
| **nC37** | nd | nd | nd | nd | nd | nd | nd |
| **nC38** | nd | nd | nd | nd | nd | nd | nd |
| **nC39** | nd | nd | nd | nd | nd | nd | nd |

Data in ppm (µg/g)

nd: not detected.

<dl: below the limit of detection

**TableS3b.** PAH composition and abundance in the initial samples.

| **Compound** | **RPW** | **RPS** | **RPCal** | **AS** | **CP** | **FdP** | **MS** |
| --- | --- | --- | --- | --- | --- | --- | --- |
| **N** | 0.11±0.029 | 0.097±0.006 | 0.049±0.009 | 0.111±0.004 | 0.27±0.03 | 0.045±0.006 | 0.06±0.03 |
| **1-MN** | <dl | <dl | 0.02±0.02 | 0.02±0.04 | 0.109±0.001 | 0.0040±0.0008 | 0.003±0.007 |
| **2-MN** | 0.005±0.009 | 0.005±0.008 | 0.02±0.01 | 0.02±0.04 | 0.305±0.003 | 0.006±0.003 | 0.005±0.004 |
| **N3** | nd | nd | 0.05±0.03 | nd | nd | nd | nd |
| **N4** | nd | nd | 0.07±0.05 | nd | nd | 0.025±0.015 | 0.001±0.002 |
| **D** | 0.09±0.05 | 0.062±0.001 | 0.12±0.08 | nd | nd | 0.005±0.003 | 0.005±0.0003 |
| **Ph** | 0.12±0.06 | 0.10±0.01 | 0.13±0.07 | nd | 0.020±0.001 | 0.004±0.003 | 0.004±0.00004 |
| **A** | 0.12±0.07 | 0.09±0.01 | 0.14±0.07 | nd | 0.024±0.007 | 0.007±0.002 | 0.0100±0.0008 |
| **Ba** | nd | nd | 0.060±0.006 | nd | nd | nd | nd |
| **Py** | 0.07±0.05 | 0.06±0.01 | 0.06±0.03 | nd | nd | 0.0047±0.0005 | 0.006±0.0005 |
| **CN** | nd | 0.009±0.001 | 0 | 0.0205±0.0009 | 0.0077±0.0005 | 0.012±0.003 | nd |
| **Fl** | nd | 0.03±0.005 | 0.04±0.08 | nd | nd | 0.0039±0.0001 | nd |
| **C** | nd | nd | 0.08±0.07 | 0.71±0.04 | nd | nd | nd |

Naphthalene (**N**), 1-methylnaphthalene (**1-MN**), 2-methylnaphthalene (**2-MN**), 1,4,5-trimethylnaphthalene (**N3**), 1,4,6,7-tetramethylnaphthalene (**N4**), dibenzothiophene (**D**), Phenanthrene (**Ph**), Anthracene (**A**), Benzo[a]anthracene (**Ba**), Pyrene (**Py**), C2-naphthalene (**CN**), Fluoranthene (**Fl**), Chrysene (**C**)

Data in ppm (µg/g)

nd: not detected.

<dl: below the limit of detection
