# Supplementary material for: Polycyclic Aromatic Hydrocarbon-Induced Changes in Bacterial Community Structure under Anoxic Nitrate Reducing Conditions
Source: Front Microbiol. 2016 Nov 8;7:1775. doi: 10.3389/fmicb.2016.01775 (PMC5099901; doi:10.3389/fmicb.2016.01775)
Supplement: Table S4 — Most probable number counts of nitrate reducing bacteria in the environmental samples. Carbon source are naphthalene (NAP), 2-methylnaphthalene (2MN), 2-naphtoic acid (2NA), anthracene (ANT), acetate (Ace), and no added carbon source (na). [file Table4.DOCX]

***Supplementary material***

**Polycyclic aromatic hydrocarbon-induced changes in bacterial community structure under anoxic nitrate reducing condition.**

Sophie-Marie Martirani-Von Abercron, Daniel Daniel, Patricia Benito-Santano, Patricia Marín and Silvia Marqués^*^

Estación Experimental del Zaidín, Department of Environmental Protection, Consejo Superior de Investigaciones Científicas, Granada, Spain.

*Author for correspondence: Silvia Marqués

Estación Experimental del Zaidín, CSIC

C/. Profesor Albareda nº1

E-18008 Granada, Spain

# [silvia@eez.csic.es](mailto:silvia@eez.csic.es)

# Tel: +34 958 181600

Fax: +34 958 129600

**Table S4.** Most probable number counts of nitrate reducing bacteria in the environmental samples. Carbon source are naphthalene (NAP), 2-methylnaphthalene (2MN), 2-naphtoic acid (2NA), anthracene (ANT), acetate (Ace) and no added carbon source (na).

| **Sample name** | **C source** | **MPN counts** | **95% C.I.** | |
| --- | --- | --- | --- | --- |
| **RPCal** | **na** | 1.95E+04 | 3.28E+03 | 8.04E+04 |
|  | **NAP** | 5.80E+03 | 1.41E+03 | 1.47E+04 |
|  | **2MN** | 2.79E+03 | 4.09E+02 | 1.03E+04 |
|  | **2NA** | 3.46E+04 | 4.98E+03 | 1.19E+05 |
|  | **ANT** | 4.27E+06 | 1.03E+06 | 1.38E+07 |
|  | **Ace** | 2.40E+07 | 4.76E+06 | 9.65E+07 |
| **RPW** | **na** | 4.27E+04 | 1.03E+04 | 1.38E+05 |
|  | **NAP** | 2.40E+07 | 4.76E+06 | 9.65E+07 |
|  | **2MN** | 7.41E+06 | 1.74E+06 | 2.15E+07 |
|  | **2NA** | 7.41E+06 | 1.74E+06 | 2.15E+07 |
|  | **ANT** | 2.40E+06 | 4.76E+05 | 9.65E+06 |
|  | **Ace** | 4.27E+07 | 1.03E+07 | 1.38E+08 |
| **RPS** | **na** | 1.43E+05 | 2.74E+04 | 6.07E+05 |
|  | **NAP** | 1.47E+07 | 2.78E+06 | 6.32E+07 |
|  | **2MN** | 9.33E+06 | 2.07E+06 | 2.71E+07 |
|  | **2NA** | 7.41E+06 | 1.74E+06 | 2.15E+07 |
|  | **ANT** | 9.33E+06 | 2.07E+06 | 2.71E+07 |
|  | **Ace** | 2.40E+08 | 4.76E+07 | 9.65E+08 |
| **AS** | **na** | 4.27E+05 | 1.03E+05 | 1.38E+06 |
|  | **NAP** | 9.18E+03 | 2.05E+03 | 2.66E+04 |
|  | **2MN** | 1.86E+02 | 3.20E+01 | 7.78E+02 |
|  | **2NA** | 9.33E+05 | 2.07E+05 | 2.71E+06 |
|  | **ANT** | 4.27E+05 | 1.03E+05 | 1.38E+06 |
|  | **Ace** | 4.27E+06 | 1.03E+06 | 1.38E+07 |
| **CP** | **na** | 7.41E+01 | 1.74E+01 | 2.15E+02 |
|  | **NAP** | 4.27E+01 | 1.03E+01 | 1.38E+02 |
|  | **2MN** | 9.33E+00 | 2.07E+00 | 2.71E+01 |
|  | **2NA** | 2.59E+03 | 3.89E+02 | 9.76E+03 |
|  | **ANT** | 2.40E+05 | 4.76E+04 | 9.65E+05 |
|  | **Ace** | 2.40E+07 | 4.76E+06 | 9.65E+07 |
| **FdP** | **na** | 1.90E+02 | 3.24E+01 | 7.89E+02 |
|  | **NAP** | 2.40E+06 | 4.76E+05 | 9.65E+06 |
|  | **2MN** | 2.04E+04 | 3.37E+03 | 8.32E+04 |
|  | **2NA** | 9.18E+03 | 2.05E+03 | 2.66E+04 |
|  | **ANT** | 7.41E+03 | 1.74E+03 | 2.15E+04 |
|  | **Ace** | 4.78E+02 | 1.16E+02 | 1.47E+03 |
| **MS** | **na** | 2.04E+00 | 3.70E-01 | 8.30E+00 |
|  | **NAP** | 4.27E+01 | 1.03E+01 | 1.38E+02 |
|  | **2MN** | 2.75E+00 | 5.90E-01 | 1.02E+01 |
|  | **2NA** | 4.27E+01 | 1.03E+01 | 1.38E+02 |
|  | **ANT** | 7.41E+01 | 1.74E+01 | 2.15E+02 |
|  | **Ace** | 9.33E+04 | 2.07E+04 | 2.71E+05 |
