# Supplementary material for: Polycyclic Aromatic Hydrocarbon-Induced Changes in Bacterial Community Structure under Anoxic Nitrate Reducing Conditions
Source: Front Microbiol. 2016 Nov 8;7:1775. doi: 10.3389/fmicb.2016.01775 (PMC5099901; doi:10.3389/fmicb.2016.01775)
Supplement: Table S6 — Significance tests performed using a two-sided Student's two-sample t-test comparing the Unifrac weighted distance. [file Table6.DOCX]

***Supplementary material***

**Polycyclic aromatic hydrocarbon-induced changes in bacterial community structure under anoxic nitrate reducing condition.**

Sophie-Marie Martirani-Von Abercron, Daniel Daniel, Patricia Benito-Santano, Patricia Marín and Silvia Marqués^*^

Estación Experimental del Zaidín, Department of Environmental Protection, Consejo Superior de Investigaciones Científicas, Granada, Spain.

*Author for correspondence: Silvia Marqués, Estación Experimental del Zaidín, CSIC, C/. Profesor Albareda nº1, E-18008 Granada, Spain, [silvia@eez.csic.es](mailto:silvia@eez.csic.es)

**Table S6.** Significance tests performed using a two-sided Student's two-sample t-test comparing the Unifrac weighted distance.

| **Samples** | **Compared group** | | **p-value** |
| --- | --- | --- | --- |
|  | **Group 1** | **Group 2** |  |
| **RPW/RPS** | initial vs. initial | initial vs. NAP | **0.001** |
|  | initial vs. initial | initial vs. 2MN | **0.005** |
| **RPCal** | initial vs. initial | initial vs. NAP | **0.05** |
|  | initial vs. initial | initial vs. 2MN | 0.10 |
|  | initial vs. initial | initial vs. HMN | 0.10 |
| **AS** | initial vs. initial | initial vs. NAP | 0.09 |
|  | initial vs. initial | initial vs. 2MN | 0.13 |
|  | initial vs. initial | initial vs. HMN | 0.17 |
| **CP** | initial vs. initial | initial vs. NAP | **0.007** |
|  | initial vs. initial | initial vs. 2MN | **0.02** |
|  | initial vs. initial | initial vs. HMN | **0.01** |
| **FdP** | initial vs. initial | initial vs. NAP | **0.02** |
|  | initial vs. initial | initial vs. 2MN | **0.01** |
|  | initial vs. initial | initial vs. HMN | **0.01** |
| **MS** | initial vs. initial | initial vs. NAP | 0.06 |
|  | initial vs. initial | initial vs. 2MN | **0.04** |
|  | initial vs. initial | initial vs. HMN | 0.08 |
