# Supplementary material for: Polycyclic Aromatic Hydrocarbon-Induced Changes in Bacterial Community Structure under Anoxic Nitrate Reducing Conditions
Source: Front Microbiol. 2016 Nov 8;7:1775. doi: 10.3389/fmicb.2016.01775 (PMC5099901; doi:10.3389/fmicb.2016.01775)
Supplement: Table S7 — Taxonomic affiliation of strains isolated on solid medium with PAHs as carbon source. [file Table7.DOCX]

***Supplementary material***

**Polycyclic aromatic hydrocarbon-induced changes in bacterial community structure under anoxic nitrate reducing condition.**

Sophie-Marie Martirani-Von Abercron, Daniel Daniel, Patricia Benito-Santano, Patricia Marín and Silvia Marqués^*^

Estación Experimental del Zaidín, Department of Environmental Protection, Consejo Superior de Investigaciones Científicas, Granada, Spain.

*Author for correspondence: Silvia Marqués, Estación Experimental del Zaidín, CSIC, C/. Profesor Albareda nº1, E-18008 Granada, Spain, [silvia@eez.csic.es](mailto:silvia@eez.csic.es)

**Table S7.** Taxonomic affiliation of strains isolated on solid medium with PAHs as carbon source.

| **(GenBank Accession No.)** | **Isolates (Sample)** | **Carbon source** | **Method** | **Closest type strain relative in GenBank database** | **Similarity (%)** |
| --- | --- | --- | --- | --- | --- |
| KX417378 | **RPCal-N 2.1** | NAP | Agar shakes | *Pseudomonas stutzeri, Pseudomonadaceae* | 99 |
| KX417379 | **RPCal-N 2.2** | NAP | Agar shakes | *Ochrobactrum anthropi, type sp., Brucellaceae* | 99 |
| KX417380 | **RPCal-N 1.1** | NAP | Agar shakes | *Paenibacillus sp, Paenibacillaceae* | 96 |
| KX417381 | **RPCal-N 1.2** | NAP | Agar shakes | *Paenibacillus sp, Paenibacillaceae* | 96 |
| KX417382 | **RPCal-2MN 1.1** | 2MN | Agar shakes | *Pseudomonas stutzeri, Pseudomonadaceae* | 99 |
| KX417383 | **RPCal-2MN 1.2** | 2MN | Agar shakes | *Pseudomonas stutzeri, Pseudomonadaceae* | 99 |
| KX417384 | **AS-2MN 1.1** | 2MN | Agar shakes | *Bacillus thioparans, Bacillaceae* | 99 |
| KX417385 | **AS-2MN 1.2** | 2MN | Agar shakes | *Bacillus thioparans, Bacillaceae* | 99 |
| KX417386 | **RPCal-N 10-2-3** | NAP | Agar plate | *Pseudomonas stutzeri, Pseudomonadaceae* | 99 |
| KX417387 | **RPCal-N 10-2-4** | NAP | Agar plate | *Pseudomonas stutzeri, Pseudomonadaceae* | 99 |
| KX417388 | **RPCal-2MN 10-2-2** | 2MN | Agar plate | *Paenibacillus graminis, Paenibacillaceae* | 97 |
| KX417389 | **RPCal-2MN 10-3-3** | 2MN | Agar plate | *Bacillus korlensis, Bacillaceae* | 98 |
| KX417390 | **RPCal-2MN 10-3-4** | 2MN | Agar plate | *Paenibacillus xylanilyticus, Paenibacillaceae* | 97 |
| KX417391 | **RPCal-HMN 10-2-3** | NAP | Agar plate | *Pseudomonas stutzeri, Pseudomonadaceae* | 99 |
| KX417392 | **RPCal-HMN 10-2-4** | NAP | Agar plate | *Pseudomonas stutzeri, Pseudomonadaceae* | 99 |
| KX417393 | **RPCal-HMN 10-2-5** | NAP | Agar plate | *Bacillus korlensis, Bacillaceae* | 99 |
| KX417394 | **RPCal-HMN10-3-2** | NAP | Agar plate | *Pseudomonas stutzeri, Pseudomonadaceae* | 99 |
| KX417395 | **RPCal-HMN 10-3-4** | NAP | Agar plate | *Pseudomonas stutzeri, Pseudomonadaceae* | 99 |
| KX417396 | **RPW-N 10-2-4** | NAP | Agar plate | *Pseudomonas stutzeri, Pseudomonadaceae* | 99 |
| KX417397 | **RPS-2MN 10-2-1** | 2MN | Agar plate | *Streptomyces sp., Streptomycetaceae* | 96 |
| KX417398 | **CP-2MN 10-3-4** | 2MN | Agar plate | *Bacillus niacini, Bacillaceae* | 97 |
| KX417399 | **CP-2MN 10-3-5** | 2MN | Agar plate | *Bacillus thermocopriae, Bacillaceae* | 98 |
| KX417400 | **CP-2MN 10-3-6** | 2MN | Agar plate | *Bacillus niacini, Bacillaceae* | 98 |
| KX417401 | **CP-HMN 10-3-3** | NAP | Agar plate | *Pseudomonas balearica, Pseudomonadaceae* | 99 |
| KX417402 | **CP-HMN 10-3-4** | NAP | Agar plate | *Bacillus korlensis, Bacillaceae* | 99 |
| KX417403 | **AS-2MN 10-2-1** | 2MN | Agar plate | *Bacillus sp., Bacillaceae* | 90 |
| KX417404 | **FdP-HMN 10-2-2** | NAP | Agar plate | *Marinilabilia sp., Marinilabiliaceae* | 98 |
| KX417405 | **FdP-2MN 10-2-3** | 2MN | Agar plate | *Bacillus sp, Bacillaceae* | 92 |
| KX417406 | **FdP-HMN 10-2-6** | NAP | Agar plate | *Marinilabilia sp, Marinilabiliaceae* | 98 |
