# Supplementary figures and images for: Polycyclic Aromatic Hydrocarbon-Induced Changes in Bacterial Community Structure under Anoxic Nitrate Reducing Conditions
Source: Front Microbiol. 2016 Nov 8;7:1775. doi: 10.3389/fmicb.2016.01775 (PMC5099901; doi:10.3389/fmicb.2016.01775)

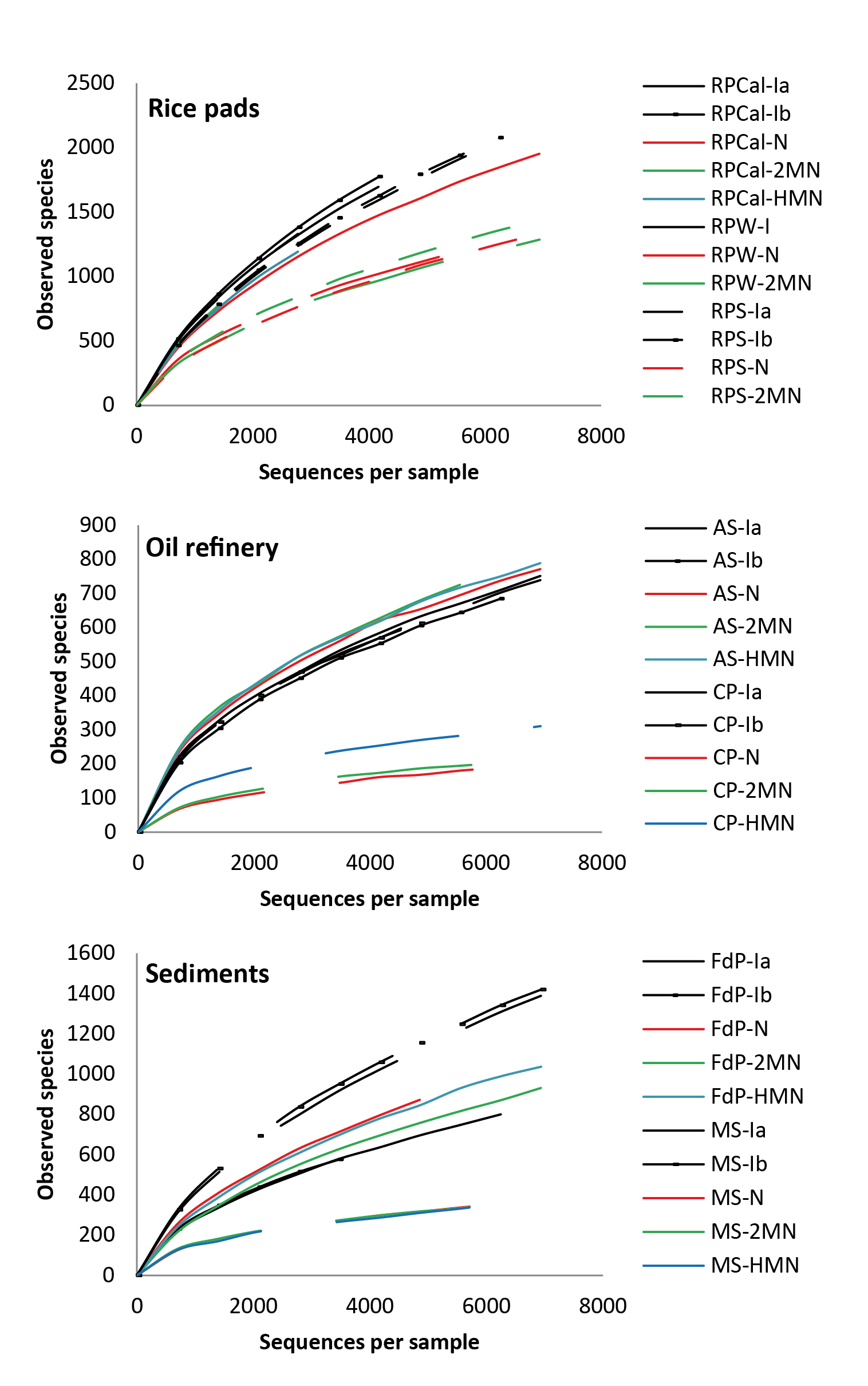

Supplement: Figure S1 — Rarefaction curves based on bacterial OTUs at a dissimilarity level of 3% of the initial samples and enrichments as calculated by Qiime. [file Image1.TIFF]

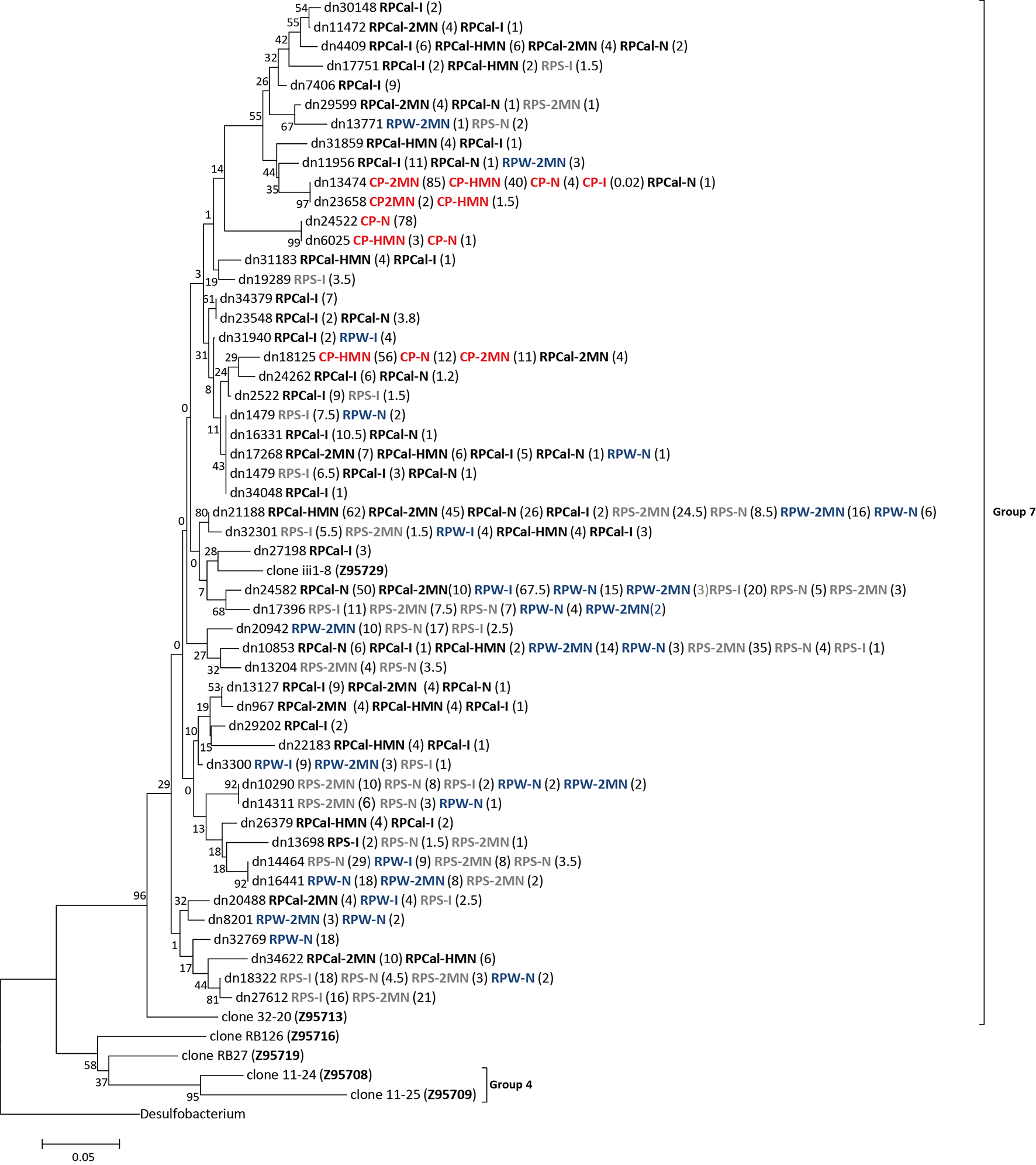

Supplement: Figure S2 — Evolutionary relationships of iii1-8 DS18 sequences retrieved from the rice-paddy and compost-pile initial sample and enrichments. The optimal Neighbour-Joining tree is shown. The bootstrap (1000 replicates) is shown next to the branches. The evolutionary distances are in the units of the number of base substitutions per site. Numbers in brackets are the percentage of each sequence type over the complete set of iii1-8 reads in each sample. The sequences of three different Acidobacteria subgroups are included (Hugenholtz et al., 1998). Numbers in brackets indicate the relative abundance (%) of each sequence in the corresponding sample. [file Image2.TIFF]

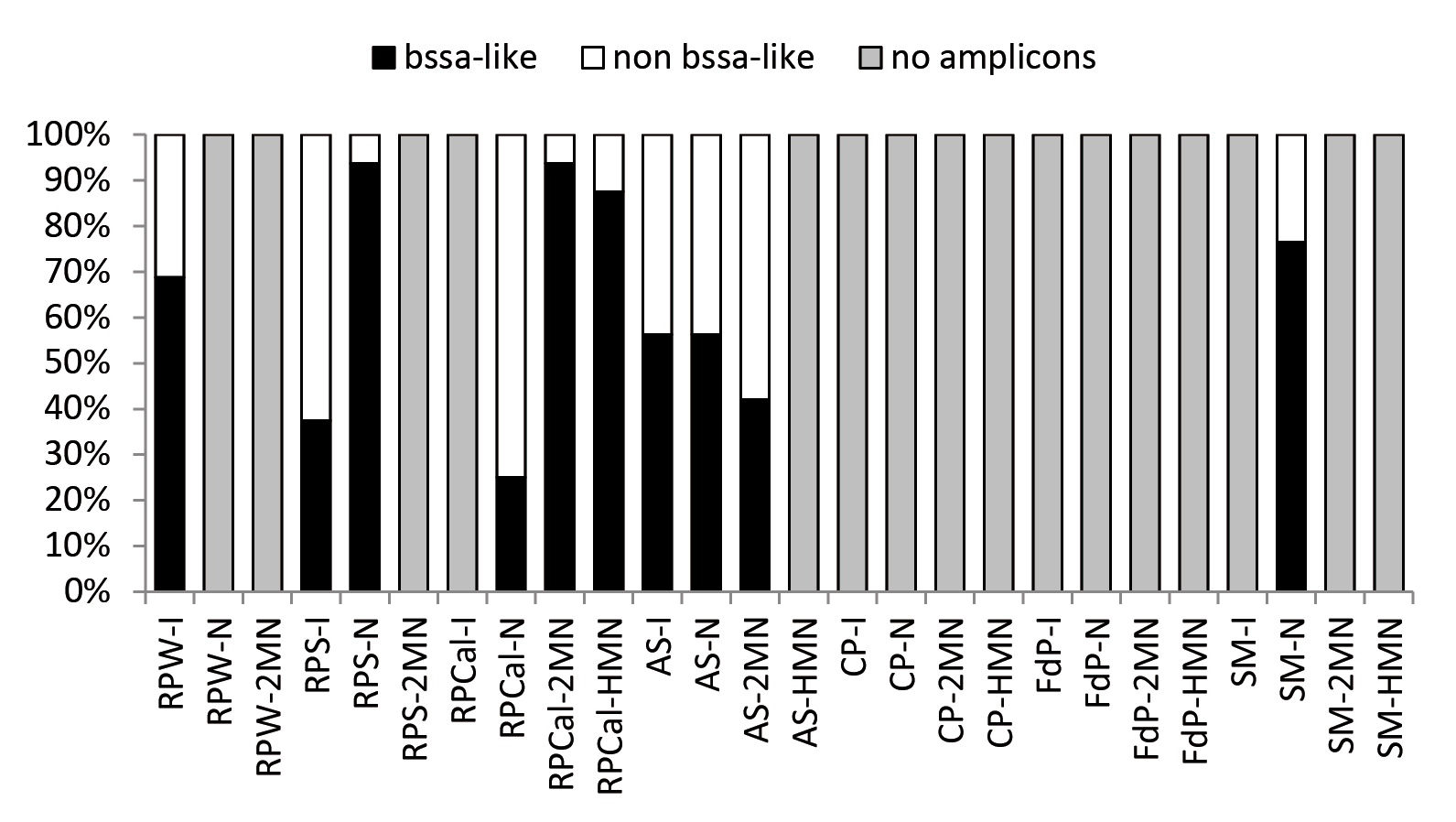

Supplement: Figure S3 — Frequency distribution of bssA-like and non-bssA-like sequence types in the different libraries constructed from the samples. [file Image3.TIFF]

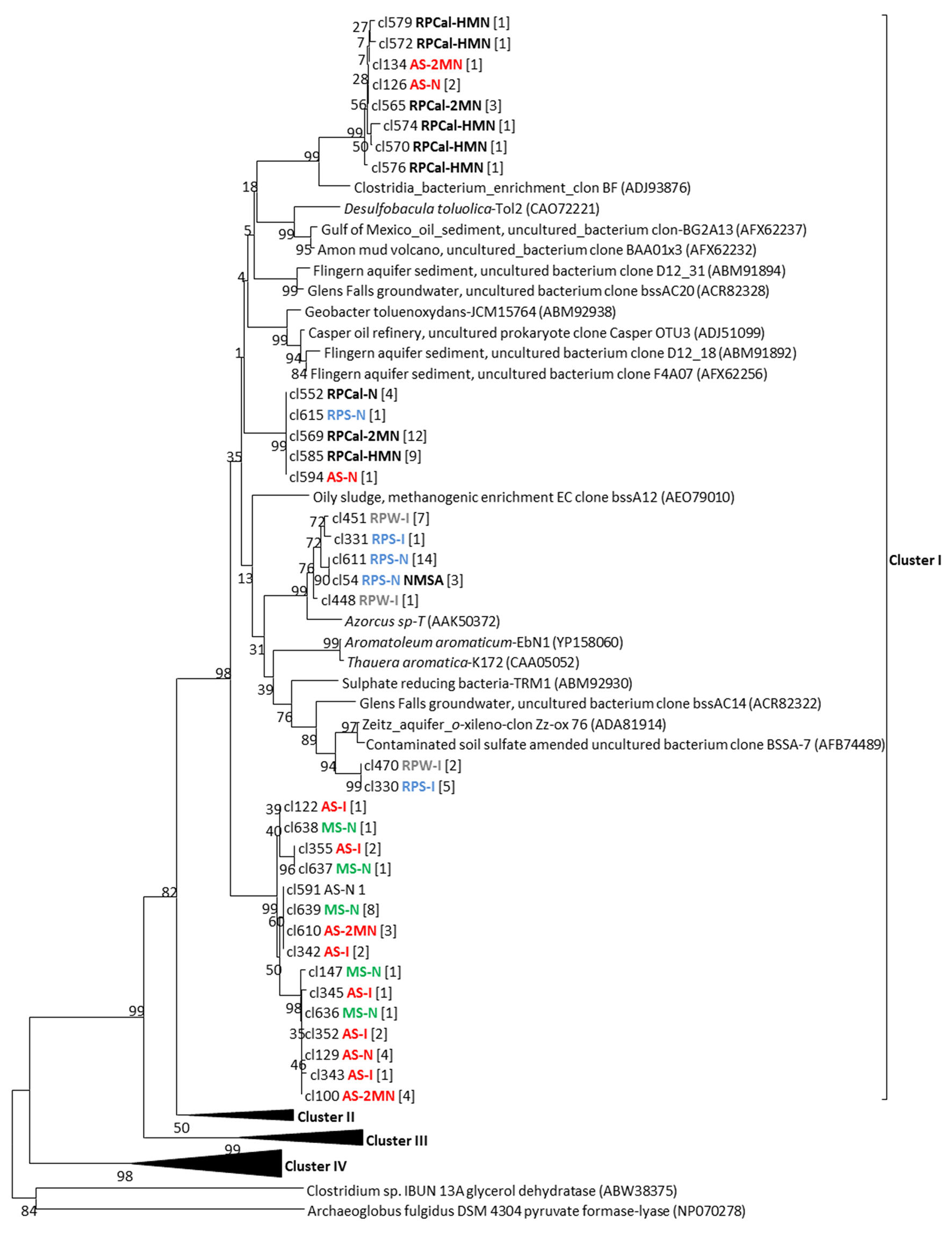

Supplement: Figure S4 — Phylogeny of partial BssA-like amino acid sequences retrieved from initial samples and enrichments. Initial samples were rice-paddy water (RPW-I; gray), rice-paddy soil (RPS-I; blue), and activated sludge (AS-I; red). Enrichments were rice-paddy soil naphthalene (RPS-N; blue), rice-paddy soil Calasparra naphthalene (RPCal-N; black), rice-paddy soil Calasparra 2MN (RPCal-2MN; black), rice-paddy soil Calasparra HMN (RPCal-HMN; black), activated sludge naphthalene (AS-N; red), activated sludge 2MN (AS-2MN; red), and marine sediments naphthalene (MS-N; green). The closest relatives, detected in anaerobic hydrocarbon-oxidizing strains or enrichments, and putative environmental sequences available in the databases at the beginning of this study are included. Cluster I includes the canonical, toluene-specific BssA sequences; cluster II includes sequences associated with the oxidation of BTEX compounds; cluster III includes the 2-methyl-naphthalene-specific nmsA sequences, putative bssA gene from the non-proteobacterial Desulfotomaculum sp. strain Ox39 and sequences retrieved from enrichments able to grow on o-xylene as the only carbon source; cluster IV comprises assA sequences. Codes in brackets are the protein GenBank accession numbers. Numbers in square brackets are the number of sequences found in the corresponding library. The tree was rooted with pyruvate formate lyase paralogs as an out-group. [file Image4.tiff]
